# Profiling Pro‐Inflammatory Proteases as Biomolecular Signatures of Material‐Induced Subcutaneous Host Response in Immuno‐Competent Mice

**DOI:** 10.1002/advs.202309709

**Published:** 2024-12-04

**Authors:** Nam M.P. Tran, Anh T.H. Truong, Dang T. Nguyen, Tram T. Dang

**Affiliations:** ^1^ School of Chemistry Chemical Engineering and Biotechnology Nanyang Technological University 70 Nanyang Drive Singapore 637459 Singapore

**Keywords:** biomaterial, host immune response, inflammation, protease, subcutaneous implant

## Abstract

Proteases are important modulators of inflammation, but they remain understudied in material‐induced immune response, which is critical to clinical success of biomedical implants. Herein, molecular expression and proteolytic activity of three distinct proteases, namely neutrophil elastase, matrix metalloproteinases, cysteine cathepsins (cathepsin‐K and cathepsin‐B) are comprehensively profiled, in the subcutaneous host response of immuno‐competent mice against different biomaterial implants. Quantitative non‐invasive monitoring with activatable fluorescent probes reveals that different microparticulate materials induce distinct levels of protease activity with degradable poly(lactic‐co‐glycolic) acid inducing the strongest signal compared to nondegradable materials such as polystyrene and silica oxide. Furthermore, protein expression of selected proteases, attributable to both their inactive and active forms, notably deviates from their activities associated only with their active forms. Protease activity exhibits positive correlations with protein expression of pro‐inflammatory cytokines tumor necrosis factor α and interleukin 6 but negative correlation with pro‐fibrotic cytokine transforming growth factor β1. This study also demonstrates the predictive utility of protease activity as a non‐invasive, pro‐inflammatory parameter for evaluation of the anti‐inflammatory effects of model bioactive compounds on material‐induced host response. Overall, the findings provide new insights into protease presence in material‐induced immune responses, facilitating future biomaterial assessment to evoke appropriate host responses for implant applications.

## Graphical abstract

Proteases are understudied in material‐induced immune responses despite their role in inflammation modulation. In immuno‐competent mice, the in vivo activity of three proteases against various subcutaneously injected microparticulate materials is profiled, demonstrating positive correlation of protease activity with protein expression of pro‐inflammatory cytokines. Effectiveness of anti‐inflammatory compounds is also predicted using protease activity as a non‐invasive indicator of pro‐inflammatory state.

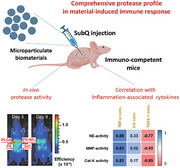

## 1 Introduction

Material‐induced host response is an immune‐mediated process that involves dynamic interaction between signaling biochemical molecules and functionally distinct immune cells, which are activated upon the implantation of medical devices or therapeutic delivery systems.^[^ ^1^, ^2^ ^]^ Inducing a favorable host response to an implanted material is critical to its eventual clinical success.^[^ ^3^ ^]^ This host response relies on the coordinated involvement of both the cellular and molecular constituents of the immune system, including immune cells, cytokines, and proteases.^[^ ^4^, ^5^, ^6^ ^]^ Consequently, gaining a comprehensive understanding of these immune constituents and their interaction would be essential to effectively modulate material‐induced host response. However, in past studies involving implanted materials, the emphasis has primarily been on the role of immune cells,^[^ ^7^, ^8^, ^9^, ^10^ ^]^ thereby leaving the molecular constituents understudied.

Thus far, cellular activities in material‐induced host response such as neutrophil recruitment and macrophage polarization are known to be regulated by cytokines, a group of extracellular small proteins.^[^ ^6^ ^]^ For example, chemokines, constituting a cytokine subset, regulate the recruitment of leukocytes to injury sites by inducing integrin expression to arrest cell rolling.^[^ ^11^ ^]^ Other major subsets of the cytokine families including proinflammatory biomolecules such as interleukin 2 (IL‐2), interleukin‐6 (IL‐6), interleukin 8 (IL‐8), and tumor necrosis factor α (TNF‐α) or anti‐inflammatory factors such as interleukin 4 (IL‐4) and interleukin 2 (IL‐2), interleukin‐6 (IL‐6), interleukin 13 (IL‐13) are key molecular mediators of both immune cell activation, recruitment, and intracellular signaling characteristic of inflammation.^[^ ^12^ ^]^

The enhancement or reduction of cytokine functions during the inflammatory cascade is regulated by proteases,^[^ ^13^ ^]^ which irreversibly break down peptide bonds through proteolytic cleavage.^[^ ^14^ ^]^ For example, the process of degrading mature TNF‐α^[^ ^15^ ^]^ and IL‐6^[^ ^16^ ^]^ into smaller and inactive fragments is carried out by neutrophil elastase and cathepsin G, leading to the suppression of neutrophil activation and inflammatory process. Moreover, various matrix metalloproteases (MMPs) cleave the truncated TNF‐α precursors to produce biologically active mature TNF‐α.^[^ ^17^ ^]^ They also decompose extracellular matrix to facilitate the migration of T lymphocytes.^[^ ^18^ ^]^

Considering this significant involvement of proteases in inflammation‐associated diseases through the regulation of cytokine activities, it is expected that they also play a major role in the material‐induced host response, which can be characterized as an inflammation‐centered continuum. However, the roles of proteases in host response to biomaterials remain poorly understood. Material‐induced protease activity has only gained more attention since the last decade along with the development of in vivo imaging techniques enabling protease activity profiling.^[^ ^19^ ^]^ Bratlie et. al. investigated the temporal changes of cathepsin activity at material‐implanted sites to establish a methodology for in vivo quantification of the material‐mediated host response.^[^ ^20^ ^]^ Liu et. al. also evaluated in vivo cathepsin activity in comparison with the effects of reactive oxygen species at biomaterial‐implanted sites.^[^ ^21^ ^]^ Assessing immune response during biomaterials‐associated infection, Daghighi et. al. quantified the activity of MMPs at implantation sites for up to 12 days post‐implantation.^[^ ^22^ ^]^ Although the correlation between increased protease expression and the extent of material‐induced host response has been established in rodent models, the assessment of protease activity in each of these published studies has been limited to a single protease type. Thus, a comprehensive investigation comparing the temporal dynamics and activity of different inflammatory proteases is instrumental in laying foundation for a deeper understanding of the role played by proteases in material‐induced host response.

Herein, we systematically investigated the dynamic evolution in the activity of inflammatory proteases from three distinct sub‐classes, namely neutrophil elastase (NE), MMPs, Cathepsins (Cathepsin K (Cat‐K) and Cathepsin B (Cat‐B)), during in vivo material‐induced host response against different materials in immuno‐competent mice. Material‐induced protease activity was evaluated by in vivo non‐invasive imaging with substrate‐based fluorescent probes in a subcutaneous implant murine model. The temporal changes in mRNA and protein expression of these proteases, along with protein expression of inflammation‐associated cytokines, were also investigated to provide new insights into their presence at the site of material implantation and their inter‐relationship. Additionally, protease activity was further investigated to assess its potential utility as a non‐invasive parameter to evaluate the anti‐inflammatory effects of bioactive compounds on material‐induced host response.

## 2 Results and Discussion

The choice of implanted materials and their associated physico‐chemical characteristics influence the extent of material‐induced inflammation during in vivo host response,^[^ ^23^ ^]^ which in turn affects the dynamics of protease activity at material‐tissue interface. This activity of proteases subsequently modulates expression of cytokines as well as infiltration and activation of immune cells.^[^ ^1^ ^]^ In this study, four different types of material formulations including polystyrene (PS), PS with terminal amino groups (PS‐NH_2_), poly(lactic‐co‐glycolic acid) (PLGA), and silica (silicon dioxide, SiO_2_) were used to induce host response in immuno‐competent SKH1‐E mice for profiling of proteases. PS is a non‐degradable polymeric material with extensive biomedical applications such as substrate for cell culture,^[^ ^24^, ^25^ ^]^ pelvic reconstruction patches,^[^ ^26^ ^]^ or candidate adjuvants to enhance immune response elicited by vaccines.^[^ ^27^, ^28^, ^29^ ^]^ We also include commercially available PS microparticles modified with amino groups (PS‐NH_2_) since the disparity in surface hydrophilicity compared to regular PS particles might result in distinct protease activity profiles. Additionally, PLGA was selected as it is a well‐known biodegradable polymer with proven clinical application for multiple FDA‐approved drug delivery systems.^[^ ^30^, ^31^ ^]^ Lastly, SiO_2,_ which has been used as drug delivery vehicles and bone‐repairing devices,^[^ ^32^, ^33^ ^]^ was chosen to represent non‐degradable inorganic biomaterials. Overall, these four different types of materials formulated as microparticles with average diameters of 5–7 µm, specifically PS (5 µm), PS‐NH_2_ (5 µm), PLGA (6.8 µm), and SiO_2_ (7 µm) (Figure S1, Supporting Information), were subcutaneously (subQ) injected on the dorsal side of hairless immuno‐competent SKH1‐E mice (Figure S2, Supporting Information). The small size of the microparticles (5–7 µm) facilitates their minimally invasive injection into the subcutaneous space through a 23G needle while the pressure caused by phosphate‐buffered saline (PBS) buffer suspending the particles is also negligible in this model.^[^ ^20^, ^21^, ^34^, ^35^ ^]^ This approach of simultaneously comparing subcutaneous biomaterial‐induced immune response of multiple formulations of polymeric microparticles in each mouse has been well‐established in published literature by the groups of Anderson et al.^[^ ^23^ ^]^ and Dang et al.^[^ ^35^ ^]^ Specifically, with up to 6 sites of material injection on the dorsal side of each mouse, the immuno‐modulatory effect of injected microparticles was previously demonstrated to be spatially localized at each injection site, with negligible influence on neighboring sites.^[^ ^20^, ^21^, ^22^, ^23^, ^34^, ^36^ ^]^

After microparticle injection, we investigated the mRNA and protein expression and activity of NE, MMPs, Cat‐K, and Cat‐B proteases in response to the subcutaneous presence of the injected materials over a 9‐day period. NE, MMPs, Cat‐K, and Cat‐B were previously identified as important biomarkers in various pathologies such as cancer or bacterial infection.^[^ ^37^, ^38^, ^39^, ^40^, ^41^ ^]^ These selected proteases, representing three types of pro‐inflammatory proteases with distinct origins, collectively orchestrate the material‐induced host response. First, NE is a serine proteinase secreted from neutrophil azurophil granules; it localizes to neutrophil extracellular traps (NETs) and acts as an antimicrobial protein to destroy pathogens and host tissues.^[^ ^42^, ^43^ ^]^ This enzyme was also shown to regulate immune cell infiltration and modulate proinflammatory cytokine expression by interacting with membrane‐associated recognition receptors.^[^ ^44^, ^45^, ^46^ ^]^ Second, MMPs are a group of zinc‐containing endopeptidases with a similar highly conserved sequence in the catalytic domain.^[^ ^47^ ^]^ Inflammation‐activated leukocytes express MMPs to mediate the destruction of extracellular matrix and modulate inflammatory processes.^[^ ^47^ ^]^ Lastly, Cat‐K and Cat‐B are two of many lysosomal cysteine proteases which are responsible for non‐specific bulk proteolysis in endosomes and lysosomes.^[^ ^48^, ^49^ ^]^ Extra‐lysosomal activities of cathepsins are often linked to ongoing pathological processes^[^ ^49^ ^]^ such as the digestion of cartilage matrix components by Cat‐K in rheumatoid arthritis^[^ ^50^, ^51^ ^]^ or osteoarthritis.^[^ ^52^, ^53^ ^]^

### 2.1 Distribution and Clearance Kinetics of Protease‐Activatable Imaging Probes in Subcutaneous Material‐Induced Host Response

Protease‐activatable imaging agents or probes have been utilized to monitor protease activity non‐invasively in animal disease models of lung injury,^[^ ^54^ ^]^ arthritis,^[^ ^55^ ^]^ and cancer.^[^ ^56^ ^]^ Though a limited number of these commercially available imaging agents have demonstrated their potential in assessing subcutaneous material‐induced host response, the distribution and clearance kinetics of most imaging agents for NE, MMPs, Cat‐K, and Cat‐B remain poorly understood. Therefore, we herein evaluated the temporal dynamics of these activatable imaging agents in SKH1‐E mice to determine the optimal time points for imaging following intravenous administration of these agents.

**Figure 1:**
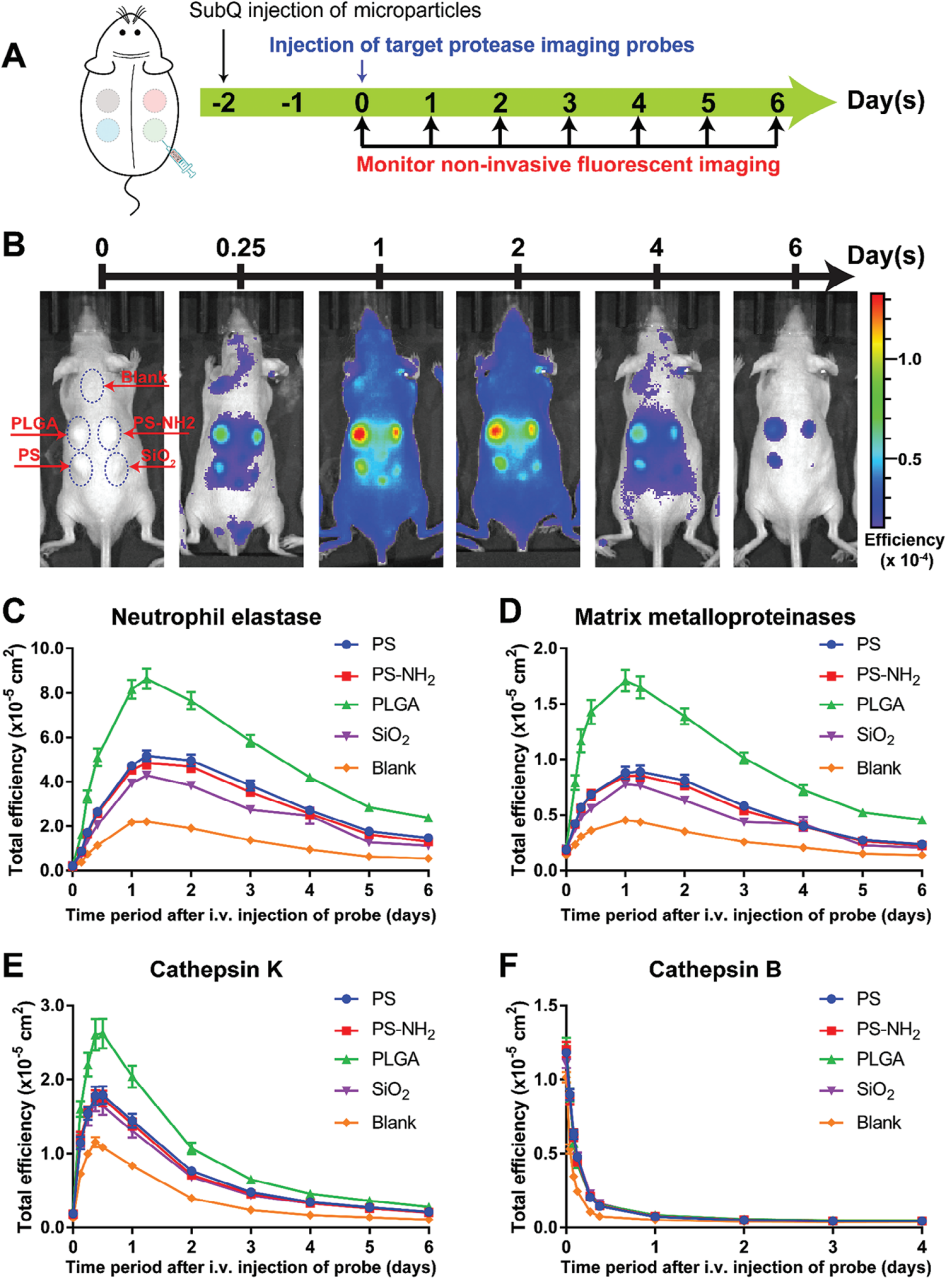
Distribution and clearance kinetics of protease‐activatable fluorescent probes in subcutaneous material‐induced host response of hairless immunocompetent SKH1‐E mice. A) Schematic of experimental design. B) Representative images of fluorescent signal on mice after intravenous injection of fluorescent probe to monitor activity of neutrophil elastase; distribution and clearance kinetics of fluorescent probe for C) Neutrophil elastase, D) MMPs, E) cathepsin K and F) cathepsin B. NE and MMPs probes were simultaneously injected via tail veins into each mouse of one group with SKH1‐E mice (N = 10). Cat‐K and Cat‐B probes were similarly administered in another group of mice (N = 9). Data represents mean ± S.E.M. of N = 10 (NE and MMPs) and N = 9 (Cat‐K and Cat‐B).

Typically, protease‐specific activatable imaging probes were intravenously administered into the mice via their tail vein one day before the desired imaging timepoint following the subQ injection of microparticles on their dorsal side (**Figure** **1**A). The probe was designed to be activated when its specific protease cleaves the peptide that linked two quenched near‐infrared fluorophores in close proximity, allowing them to diffuse apart and become brightly fluorescent.^[^ ^57^ ^]^ The generated fluorescent signal was monitored non‐invasively following the timeline presented in the schematic of Figure 1A. The excitation and emission wavelengths of imaging probes for NE and Cat‐K are 675 and 720 nm, respectively and those of MMPs and Cat‐B are 745 and 800 nm, respectively. Since there are 2 different windows of excitation and emission wavelengths for these probes, each mouse received simultaneously a pair of NE^[^ ^54^ ^]^ and MMPs^[^ ^56^ ^]^ probes or a pair of Cat‐K^[^ ^55^ ^]^ and Cat‐B.^[^ ^57^ ^]^ Representative fluorescent images in Figure 1B demonstrated the temporal variation in signal of the NE activatable probe following its intravenous administration. The NE‐activated fluorescent signals induced by each of the four injected material formulations peaked simultaneously at 24 h following probe administration and gradually declined at all injection sites until returning to the background level after 6 days. Furthermore, the fluorescent signals captured at the injection sites of all four materials exhibited a similar trend of dynamic variation, confirming the consistent activation of protease‐activatable probes across all four microparticle injection sites in the same mouse. Based on the quantification of fluorescent signal from the protease‐specific imaging probes, maximum activity of NE and MMPs (Figure 1C,D, respectively) was recorded at 24 h following probe administration, and more than 80% of the signal was cleared off on day 6. Interestingly, Cat‐K imaging signal reached its maximum at 9 h following probe administration but reduced rapidly with only 20% of this peak value remaining after 3 days (Figure 1E).

In separate in vitro experiments, we also verified that the commercially sourced imaging probes could be activated by their corresponding proteases (Figure S3A(i),B(i),C(i),D(i), Supporting Information). In vitro analysis confirmed that fluorescent signal acquired by incubating human recombinant MMP‐9 or NE with its corresponding protease exhibited a positive non‐linear relationship with enzyme concentration (Figure S4, Supporting Information). Similar increase in fluorescent signal was observed when these imaging probes were incubated with microparticle‐containing subQ tissue retrieved from the dorsal region of mice (Figure S5, Supporting Information), demonstrating the ex vivo activity of the proteases in the retrieved tissue (Figure S3A(ii),B(ii),C(ii),D(ii), Supporting Information). In parallel in vitro experiments, the interaction between either PS or PS‐NH_2_ microparticles with imaging probes resulted in an increase in fluorescent signal despite the absence of the proteases or retrieved tissues (Figure S6, Supporting Information). However, this signal artifact was not observed when the retrieved subQ tissues containing microparticles were exposed to the imaging probes (Figure S3E(iii),F(iii),G(iii),H(iii), Supporting Information). Similarly, this signal artifact was also suppressed in vitro when bovine serum albumin (BSA) was added to the mixture of microparticles and imaging probes (Figure S7, Supporting Information). In a separate experiment, we also demonstrated that there was no differential adsorption of each imaging probe by the four microparticle formulations used in this study (Figure S8, Supporting Information). These findings suggested that signal artifact due to material‐probe interaction would be suppressed in vivo by the abundant presence of proteins, particularly albumin, at the material implant sites.

In contrast to the gradual accumulation and clearance kinetics observed for NE, MMPs, and Cat‐K imaging probes, the Cat‐B agent rapidly circulated throughout the mouse body, resulting in high fluorescent signals across the entire dorsal region immediately after intravenous injection of the probe (Figure S9, Supporting Information; 0 h after probe injection). Thereafter, the signals faded rapidly in all locations on the animal body including the sites of microparticle injection as well as the skin region that was not injected with any particles (Figure 1F; Figure S9, Supporting Information). As reported by Lin et al., this agent reached its maximum activation within six to 8 h after its intravenous administration in apoE^−/−^ female mouse model of atherosclerosis.^[^ ^57^ ^]^ However, the data in our study indicates that this Cat‐B probe was unable to produce the signals in targeted subcutaneous locations containing injected microparticles on the dorsal side of immunocompetent male SKH1‐E mice. Thus, we excluded the assessment of this probe and its corresponding protease, Cat‐B, in subsequent experiments.

### 2.2 Temporal Dynamics of Protease Activity in Subcutaneous Material‐Induced Host Response

**Figure 2:**
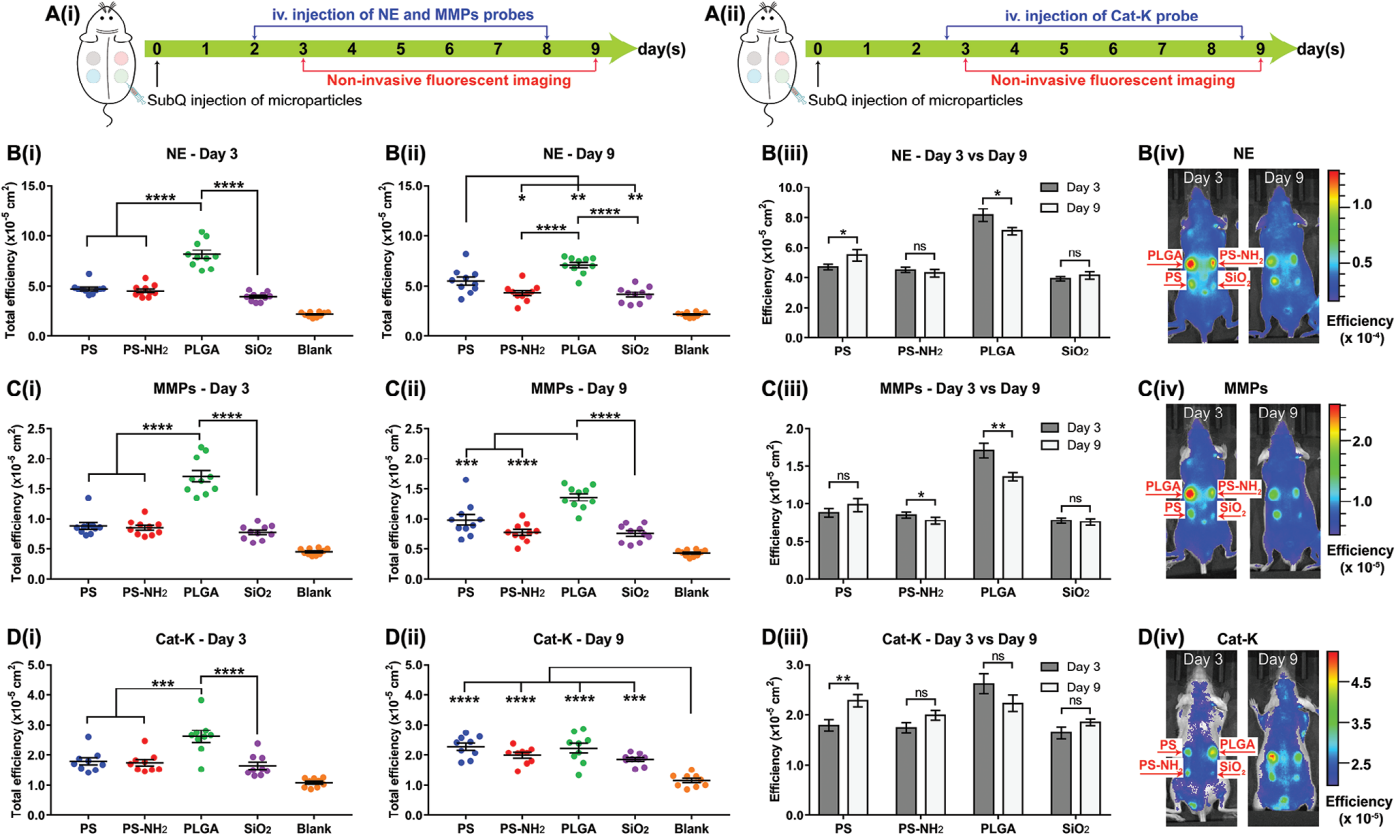
Temporal dynamics of protease activity during the early stage of material‐induced host response. Schematic of experimental design for A(i)) MMPs and NE probes and A(ii)) Cat‐K probe. Fluorescent signal quantified from imaging probes of B) NE, C) MMPs, and D) Cat‐K; fluorescent signal at day 3 of B(i)) NE, C(i)) MMPs, D(i)) Cat‐K, and at day 9 of B(ii)) NE, C(ii)) MMPs, D(ii)) Cat‐K; comparison B(iii), C(iii),D(iii)) of fluorescent signals and representative images B(iv),C(iv),D(iv)) on day 3 versus day 9. Data represents mean ± S.E.M. of N = 10 (NE and MMPs) and N = 9 (Cat‐K). *p*‐values of B3, C3, D3 were determined by paired 2 tailed *t*‐test. *p*‐values of B(i), C(i), D(i), B(ii), C(ii), D(ii) were determined by one‐way ANOVA with Tukey's multiple comparison test. (^*^), (^**^), (^***^), (^****^), and ns denote *p* < 0.05, *p* < 0.01, *p* < 0.001, *p* < 0.0001, and non‐significant, respectively.

During material‐induced host immune response, the dynamics of protease activity might contribute to the modulation of cellular infiltration by myeloid cells and lymphocytes to the host‐material interface. This temporal dynamic of protease activity at implant proximity might vary in the presence of different types of material, each with distinct physico‐chemical characteristics. Therefore, we herein investigated the temporal dynamics of protease activity during the in vivo host response to four different material formulations after their subQ injection into immuno‐competent SKH1‐E mice. Protease activity at day 3 and 9 was monitored based on the fluorescent signals generated from protease‐activatable imaging agents, which were administered and monitored following the schematic timeline shown in **Figure** **2**A(i) for NE and MMPs activities and Figure 2A(ii) for Cat‐K activity.

Overall, the combined data in Figure 2B–D showed that the fluorescent signals indicating activities of NE, MMPs, and Cat‐K at the injection sites of all four materials were more intense than at the control sites (blank) without injected materials. Furthermore, on day 3, different material formulations induce signals of varied intensities reflecting distinct levels of activity for each protease (Figure 2B(i)–D(i)). Specifically, PLGA microparticles induced the strongest signals at day 3 compared to that of PS, PS‐NH_2_, and SiO_2_ microparticles when monitored with each of the three protease‐activatable imaging probes (Figure 2B(i)–D(i)). The only deviation from this trend was the similar levels of Cat‐K activity for all four materials on day 9 after particle injection (Figure 2D(ii)).

We further observed that the activity of each protease had distinct patterns of temporal changes in response to different types of materials. First, the NE‐activatable imaging probe was highly fluorescent at the PLGA injection sites on day 3 (Figure 2B(i)) but its signal was significantly dampened on day 9 (Figure 2B(ii,iii)). In contrast, NE activities were equally low at the sites containing PS, PS‐NH_2_, and SiO_2_ on day 3 (Figure 2B(i)). From day 3 to 9, the NE signal induced by PS increased (Figure 2B(iii)) while the signals for PS‐NH_2_ and SiO_2_ remained unchanged (Figure 2B(iii)). Similarly, MMPs activity at the PLGA injection site was highest on day 3 (Figure 2C(i)) but decreased on day 9 (Figure 2C(iii)). From day 3 to 9, the signal for MMPs activity at PS‐NH_2_ also exhibited decreasing trend (Figure 2C(iii)) whereas MMPs activity remained unchanged at PS and SiO_2_ sites during this time period (Figure 2C(iii)). Lastly, for Cat‐K activity, only PS‐induced signal was significantly increased on day 9 compared to day 3 while Cat‐K activity due to PLGA, PS‐NH_2_, and SiO_2_ remained unchanged (Figure 2D(iii)). These results indicated that NE, MMPs, and Cat‐K were differentially regulated at the material injection sites by the host immune system depending on the types of materials presents.

Even though the activity of each protease and its temporal changes could be compared across different materials, it was not possible to compare expression of different proteases induced by the same material. The measured peak fluorescent signals of the probe distribution kinetics graph in Figure 1 depends not only on the activity of the protease but also other parameters such as the amount of fluorophores, the radiance per fluorophore and the time of imaging measurement after probe injection. Thus, this study focused solely on comparing the activity of the same protease across different materials by using the same probe in any comparative analysis. Specifically, for each probe, the radiance per fluorophore and the time point of measurement were kept constant while the measured fluorescent signal was assumed to depend primarily on the amount of activated fluorophores, which positively correlates with the activity of the protease of interest (Figure S4, Supporting Information).

### 2.3 mRNA Expression of MMPs Family in Subcutaneous Material‐Induced Host Response

The temporal dynamics of protease activity in response to different materials was analyzed based on the fluorescent signal generated by protease‐specific activable probes as shown in Figure 2. The NE and Cat‐K fluorescent signals in Figure 2B,D were specifically generated by the activity of proteases NE and Cat‐K, respectively.^[^ ^54^, ^55^ ^]^ However, the imaging signal of MMPs in Figure 2C could be collectively contributed by the activities of multiple proteases in the MMPs family such as MMP2, MMP3, MMP7, MMP9, MMP12, and MMP13 with MMP9 being most dominant.^[^ ^56^ ^]^ Each protease in the MMPs family has some variation in substrate specificity that determines their role in extracellular matrix (ECM) breakdown and wound healing. Specifically, interstitial collagenases comprising MMP1, MMP8 and MMP13 preferentially cleave type I, II, and III collagen while gelatinases such as MMP2 and MMP9 act more effectively on collagen type IV and V.^[^ ^58^ ^]^ Thus, it is important to determine the subclasses in this MMPs family, if any, that specifically contributed to the MMP activity during material‐induced host response as shown in Figure 2C.

Therefore, to investigate the effect of material presence on mRNA expression of different MMPs at implantation sites, we quantified mRNA expression corresponding to MMP2, MMP3, MMP7, MMP9, MMP12, and MMP13 genes by RT‐qPCR analysis of the mouse subQ tissues containing microparticles retrieved on day 3 and day 9 following microparticle injection (Figure S10, Supporting Information). First, the mRNA expressions for all subQ tissues containing microparticles were normalized to house‐keeping mRNA glyceraldehyde 3‐phosphate dehydrogenase (GAPDH) (Figure S11, Supporting Information). Overall, amongst the investigated MMPs, MMP12 expressed the highest relative mRNA levels normalized against GAPDH in all subQ tissues containing PS, PS‐NH_2_, PLGA, or SiO_2_ particles at day 3, reaching more than 14% (Figure S11A, Supporting Information). Relative mRNA levels of MMP9 and MMP13 were low, in the range from about 0.0% to 1.5% while those of MMP2 and MMP3 were from 1.3% to 4.4% at day 3 following particle injection. Only the relative mRNA expression level for MMP7 at day 3 was lower than 0.01% (Figure S11A, Supporting Information), and MMP7 mRNA level for day 9 samples were not detectable (Figure S11B, Supporting Information). Thus, we excluded MMP7 in subsequent analysis.

**Figure 3:**
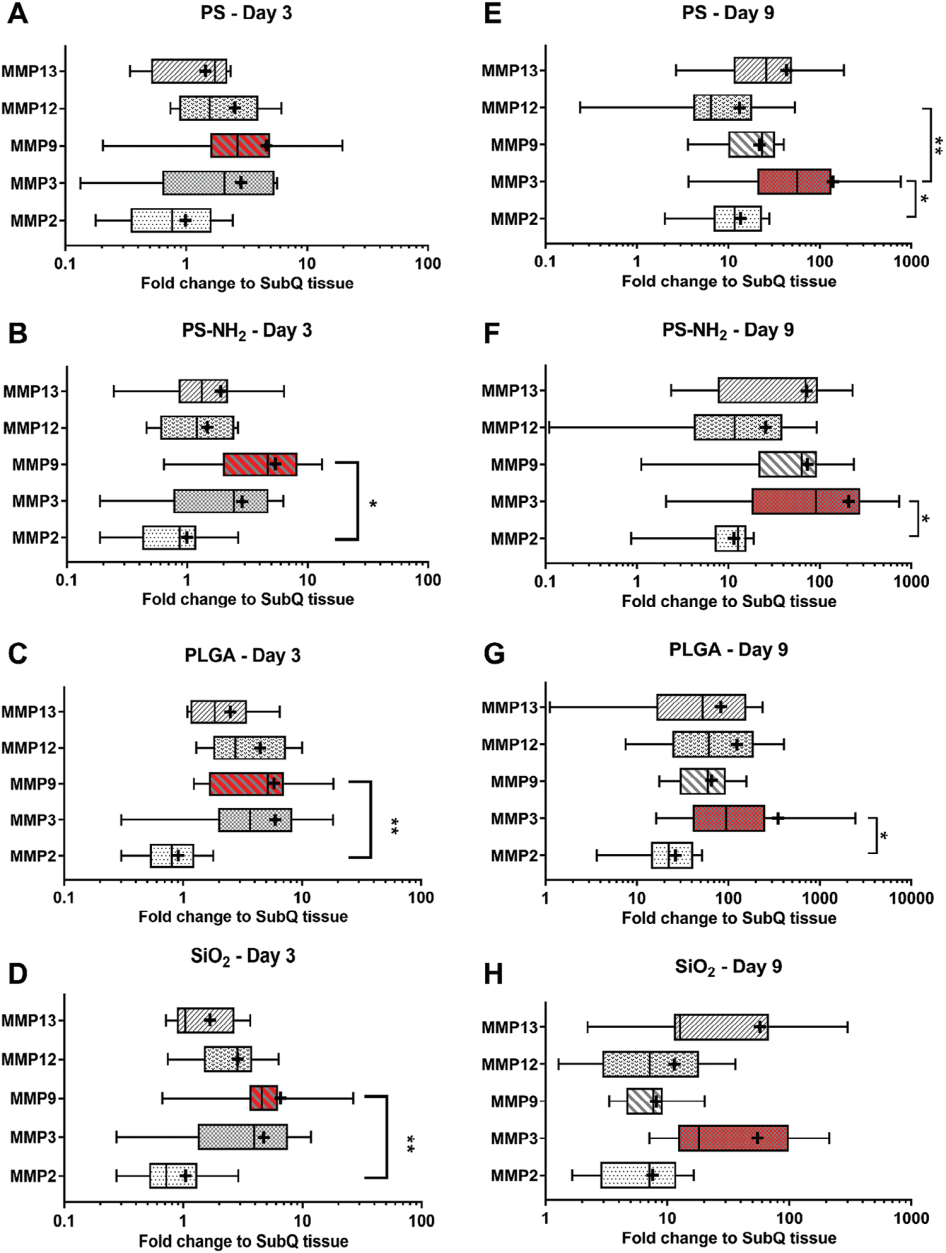
Fold change of mRNA expression for individual MMP proteases in SKH1‐E mice during material‐induced host response to A,E) PS, B,F) PS‐NH_2_, C,G) PLGA, and D,H) SiO_2_ on (A–D) day 3 or (E–H) day 9 following material injection. MMP9 and MMP3 mRNA expression were more sensitive to material‐induced upregulation on day 3 and 9, respectively. Boxes represent medians ± IQR (interquartile range). Whiskers represent the minimum and maximum observations. “+” sign represents mean of data. *p*‐values were determined by the non‐parametric test (Kruskal–Wallis test) followed by multiple comparison Dunn's test. ^*^ *p* < 0.05, ^**^ *p* < 0.01, ^***^ *p* < 0.001. N = 9 injection repeat for PS‐Day 3 and SiO_2_‐Day 9 and N = 10 for other material at each time point.

Next, we further analyzed the relative fold change of mRNA expression for MMP2, MMP3, MMP9, MMP12, and MMP13 from all material‐tissue samples (PS, PS‐NH_2_, PLGA, and SiO_2_) with reference to control subQ tissue retrieved from the dorsal region without injected microparticles (**Figure** **3**; Figure S12, Supporting Information). Generally, the mRNA expressions of most selected MMPs genes at the microparticle injection sites increased on day 9 compared to those on day 3 (Figure S12, Supporting Information). Notably, PLGA and SiO_2_‐containing tissues at day 3 had significantly higher relative fold change of mRNA expression for MMP9 than that for MMP2 or MMP13 (Figure 3C,D). However, on day 9, the fold change of MMP3 mRNA expression was significantly higher than that of MMP2 mRNA in tissues containing PS, PS‐NH_2_, and PLGA microparticles (Figure 3E–G). We observed that the mean fold change values of MMP9 and MMP3 mRNA at all injected material samples were highest compared to those of other MMPs mRNA at day 3 and 9, respectively (Figure 3) albeit without statistical difference. Therefore, we postulated that mRNA expression of MMP9 was the most sensitive to material‐induced upregulation amongst all members of the MMPs family at the early time point of day 3, while MMP3 was the most upregulated at the later time point of day 9. This temporal dynamics in mRNA expression of MMPs (Figure 3) correlated with reported function of MMP9 primarily during the initial step of early neutrophil recruitment^[^ ^59^ ^]^ and that of MMP3 during the later remodeling process of connective tissues.^[^ ^60^ ^]^ Thus, we focused on MMP9 and MMP3 proteins in subsequent protein expression experiments to understand how these metalloproteases were regulated at the sites of material implants.

### 2.4 Protein Expression of Proteases in Subcutaneous Material‐Induced Host Response

**Figure 4:**
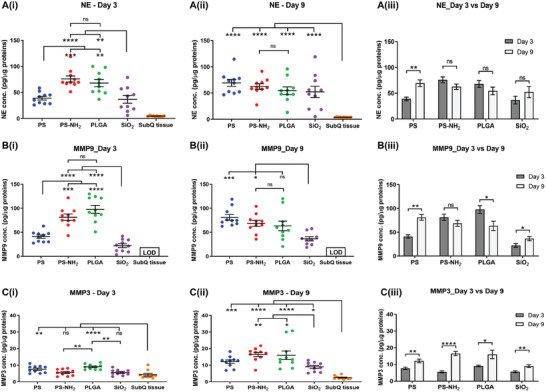
Protease protein expression from retrieved subQ tissues containing injected materials. Normalized protein concentration of targeted proteases A(i–iii)) neutrophil elastase, B(i–iii)) MMP9, C(i–iii)) MMP3 in 1 µg total protein of each retrieved subQ tissue containing microparticles. Data represents mean ± S.E.M. of N = 10. *p*‐values of A(i, ii),B(i, ii),C(i, ii) were determined by one‐way ANOVA with Tukey's multiple comparison test. *p*‐values of A3, B3, C3 were determined by non‐paired 2 tailed *t*‐test. MMP9 protein concentration of subQ tissue on day 9 was lower than limit of detection (LOD). (^*^), (^**^), (^***^), and (^****^) denote *p* < 0.05, *p* < 0.01, *p* < 0.001, and *p* < 0.0001, respectively.

We further evaluated protein‐level expression of these proteases to capture the presence of both their pro‐ and mature forms and investigate the relationship between protease activity and protein expression. Specifically, we quantified protein expression of NE, MMP3, MMP9 present in the lysate extracted from the retrieved subQ tissues containing injected microparticles on day 3 and day 9 after material injection. **Figure** **4** showed that when compared to control subQ tissue, all four materials eventually caused upregulation in protein expression of the proteases at the sites where they were injected. At day 3, NE and MMP9 protein expressions were upregulated at all material‐injected sites compared to control subQ tissue (Figure 4A(i),B(i)) while the expression of MMP3 protein was only upregulated in response to PS and PLGA (Figure 4C(i)). On day 9, all material‐injected sites contained more NE, MMP9, and MMP3 proteins than subQ tissue (Figure 4A(ii)–C(ii)).

However, the normalized protein expression of each protease at the injection sites (Figure 4) did not always correlate with the protease activity measured by fluorescent signals from the activatable imaging probes (Figure 2). Specifically, some consistency was observed as indicated by the highest protein expression of NE and MMP9 induced by PLGA on day 3 post‐injection (Figure 4A(i),B(i)), matching the strongest fluorescent signals representing protease activities of these two proteases at PLGA injection site (Figure 2B(i),C(i)). In contrast, NE and MMPs protease activities at PS‐NH_2_ injection sites were lower than that at PLGA sites at both time points (Figure 2B(i,ii),2C(i,ii)). However, the normalized protein expressions of NE and MMP9 at PS‐NH_2_ sites were similar to PLGA sites on both day 3 and day 9 post microparticles injection (Figure 4A(i,ii),B(i,ii)).

Similarly, during this early stage of the host response to the material, there was notable deviation between the protein expression of individual MMPs (Figure 4) and their corresponding mRNA levels (Figure 3). Specifically, the normalized concentration of MMP3 protein was significantly lower than the normalized concentration of MMP9 protein in all material‐contained tissues on both day 3 (Figure S13A, Supporting Information) and on day 9 after material injection (Figure S13B, Supporting Information). In contrast, MMP3 mRNA relative expression was dominant compared to MMP9 mRNA on both day 3 (Figure S11A, Supporting Information) and day 9 (Figure S11B, Supporting Information). Furthermore, from day 3 to 9, MMP9 protein expression increased at PS and SiO_2_ injection sites but remained unchanged at PS‐NH_2_ site and decreased at PLGA site (Figure 4B(iii)). In contrast, MMP9 mRNA significantly increased from day 3 to 9 at the injection sites of PS, PS‐NH_2_, and PLGA but remained unchanged at SiO_2_ site (Figure S12, Supporting Information).

Moreover, although protein level of all three proteases NE, MMP9, and MMP3 were upregulated in material‐induced host response compared to blank SubQ tissue without injected materials (Figure 4), their protein concentrations were differentially expressed depending on the type of materials involved. NE proteins were expressed more abundantly at PLGA and PS‐NH_2_ sites compared to PS and SiO_2_ sites at day 3 (Figure 4A(i)). However, at day 9, the NE protein concentrations were similar for all retrieved tissues containing all four types of material (Figure 4A(ii)). A similar trend was observed for MMP9 protein expression with PS‐NH_2_ and PLGA microparticles induced a higher amount of MMP9 protein than PS and SiO_2_ microparticles at the early time point of day 3 (Figure 4B(i)). However, PLGA sites and SiO_2_ sites induce the least amount of MMP9 protein on day 9 (Figure 4B(ii)). Lastly, MMP3 protein expression was increased in response to PLGA microparticles compared to the other two materials at day 3 (Figure 4C(i)). At day 9, its protein expression was comparable across PLGA, PS and PS‐NH_2_ sites, while SiO_2_ site induced the least MMP3 protein (Figure 4C(ii)).

Evidently, the types of material also affected the temporal changes in the protein expression of each proteases. From day 3 to 9, protein level of NE increased significantly at PS sites (Figure 4A(iii)). However, this increase was not statistically evident in mRNA expression of NE (Figure S14, Supporting Information). MMP9 protein expression increased at the injection sites of PS and SiO_2_ over the same duration but decreased significantly at PLGA sites (Figure 4B(iii)). On the other hand, MMP3 protein at all materials‐containing sites was significantly increased at day 9 compared to day 3 (Figure 4C(iii)). Particularly, compared to the amount of MMP3 protein induced by PS‐NH_2_ on day 3, the equivalent value on day 9 increased approximately three‐fold (Figure 4C(iii)). This notable increase in MMP3 protein expression was not observed for other proteases NE and MMP9 at injection sites of all materials (Figure 4A(ii),B(iii)).

Lastly, protein expression of Cat‐K protease was not quantifiable from the retrieved subcutaneous tissues, presumably due to a negligible concentration of Cat‐K which was below the detection limit of the Cat‐K ELISA assay. RT‐qPCR analysis of the transcriptional expression of Cat‐K mRNA from the retrieved material‐containing tissues showed that all material‐containing tissues had significant upregulation in Cat‐K mRNA from day 3 to 9 (Figure S15, Supporting Information). The presence of Cat‐K mRNA corroborates the activity measured with Cat‐K activatable probe (Figure 1E) despite the challenge of detecting Cat‐K protein expression with ELISA.

### 2.5 Correlation Analysis of Protease Activity and Protein Expression of Inflammation‐Associated Cytokines in Subcutaneous Material‐Induced Host Response

**Figure 5:**
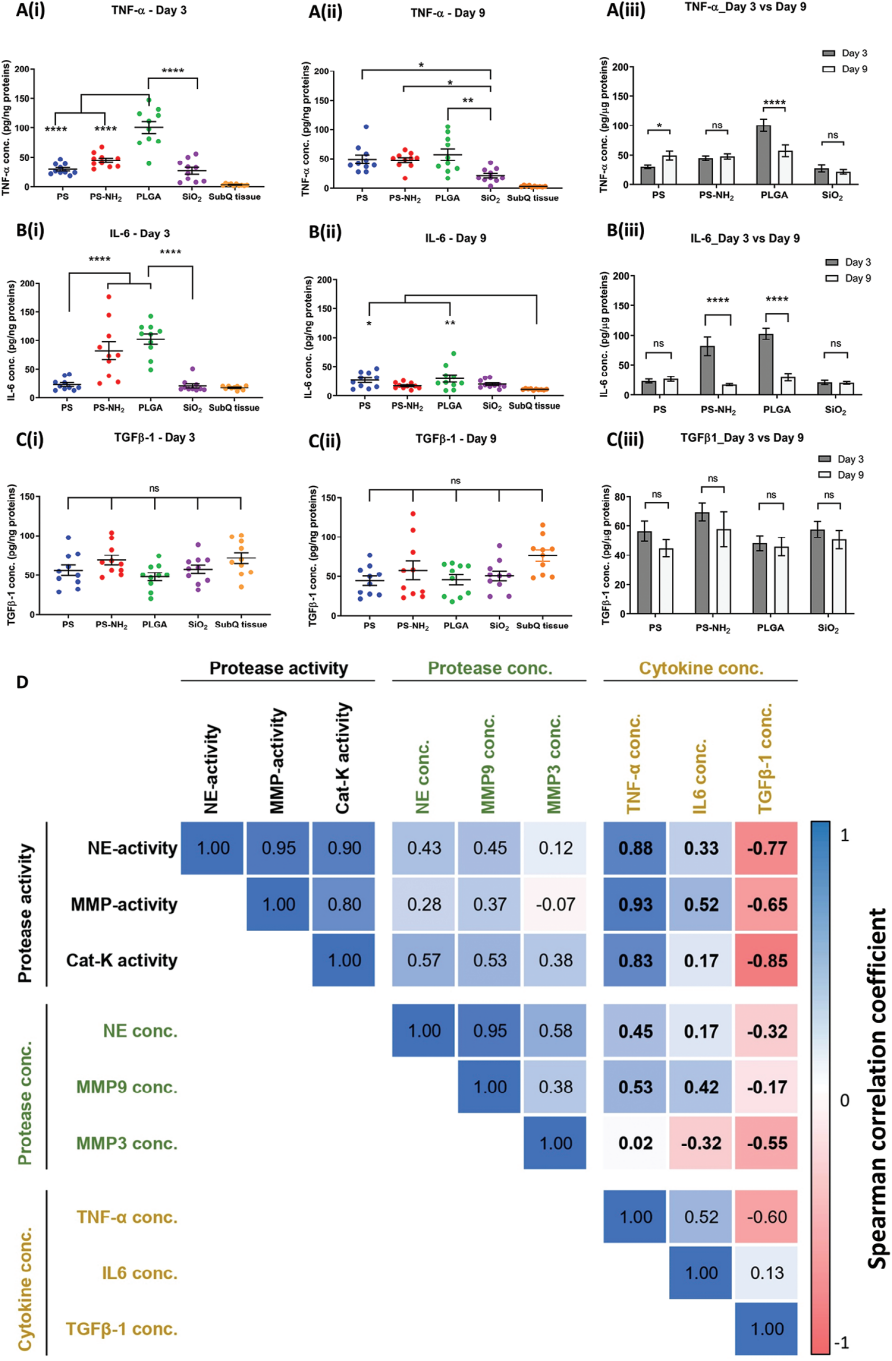
Correlation analysis of protease activity and protease protein concentration with protein expression of inflammation‐associated cytokines from microparticle‐containing subQ tissues. Normalized protein concentration of pro‐inflammatory cytokines A(i–iii)) TNF‐α, B(i–iii)) IL‐6 and pro‐fibrotic cytokine C(i–iii)) TGF‐β1 in microparticle‐containing subQ tissues retrieved on days 3 and 9. Data represents mean ± S.E.M. of N = 10. *p*‐values of A(i, ii),B(i, ii),C(i, ii) were determined by one‐way ANOVA with Tukey's multiple comparison test. *p*‐values of A3, B3, C3 were determined by non‐paired 2 tailed *t*‐test. (^*^), (^**^), (^***^), and (^****^) denote *p* < 0.05, *p* < 0.01, *p* < 0.001, and *p* < 0.0001, respectively. D) Spearman correlation coefficient matrix of 9 variables including protease activity (NE‐activity, MMP‐activity, CatK‐activity), protease protein expression (NE conc., MMP9 conc., MMP3 conc.) and cytokine protein expression (TNF‐α conc., IL‐6 conc., TGF‐β1 conc.). The number within each cell of the matrix indicates the Spearman correlation coefficient between each pair of variables out of 9 variables analyzed for 10 sets of samples. Color intensity of each cell denotes the magnitude of the corresponding correlation efficient with positive correlations shown in blue tone and negative correlations shown in red tone.

Next, we evaluated the potential utility of protease activity as a non‐invasive parameter indicative of the pro‐ or anti‐inflammatory state of the microenvironment at the microparticle injection sites. Specifically, to determine if any correlation exists between protease profile with the presence of other immune regulators, we further quantified the protein expression of key inflammation‐associated biomarkers including TNF‐α, IL‐6 and TGF‐β1, in lysate samples extracted from retrieved subcutaneous tissues containing injected microparticles on days 3 and 9 post‐injection (**Figure** **5**A–C). TNF‐α and IL‐6 are primarily pro‐inflammatory biomarkers,^[^ ^61^, ^62^, ^63^ ^]^ whereas TGF‐β1 has pleiotropic effects on inflammation, including resolving inflammation and facilitating wound healing while also promoting fibrosis.^[^ ^64^, ^65^ ^]^

First, the protein expression of the two pro‐inflammatory cytokines TNF‐α and IL‐6 was differentially induced depending on the type of microparticle formulations evaluated, with PLGA microparticles induced the highest expression of both cytokines at day 3 post‐implantation (Figure 5A(i),B(i)). Moreover, as expected, compared to blank subQ tissues, the majority of materials induced higher expression of these cytokines at both timepoints (Figure 5A(i,ii),B(i,ii)). Furthermore, except for PS, which induced increased TNF‐α expression from day 3 to 9 (Figure 5A(iii)), the expression of TNF‐α and IL‐6 for the remaining materials either decreased or remained unchanged from day 3 to 9 (Figure 5A(iii),B(iii)). In contrast, there was no significant difference in the levels of TGF‐β1 in blank subQ tissues compared to tissues injected with microparticles at both timepoints (Figure 5C(i,ii)). Furthermore, the protein expression of TGF‐β1 remained constant between the two timepoints for all material groups, as well as with blank subQ tissues (Figure 5C(iii)).

To quantitatively analyze the correlation between expression of inflammation‐associated cytokines (TNF‐α, IL‐6 and TGF‐β1), protease activity (NE, MMPs, and Cat‐K) and protease protein expression (NE, MMP3 and MMP9), we analyzed the Spearman correlation coefficient for each pair of these biological variables. The results, presented as the Spearman correlation coefficient matrix in Figure 5D, suggest that protease activity (NE, MMPs, and Cat‐K) exhibited positive, monotonic correlations with the expression of both pro‐inflammatory cytokines in material‐induced host responses, with notably higher coefficients of 0.83‐0.93 for TNF‐α compared to equivalent values of 0.17–0.52 for IL‐6. In comparison, protease protein concentration primarily exhibited weaker positive, monotonic correlations with expression of both pro‐inflammatory cytokines with the exception of MMP3 having negative correlation with IL‐6. In contrast, protease activity exhibited a notably stronger negative, monotonic correlation with the expression of pro‐fibrotic cytokine TGF‐β1 while protease protein concentration exhibited a weaker negative, monotonic correlation with the same cytokine. Overall, these findings demonstrate that protease activity simultaneously has a strong positive, monotonic correlation with the expression of the key pro‐inflammatory cytokine TNF‐α and a negative correlation with the pro‐fibrotic cytokine TGF‐β1.

We also attempted to evaluate whether protease activity measured at the early time points herein has an implication to fibrotic response against injected microparticles in the later stage of material‐induced host response. Obtained by histological analysis of the sectioned material‐containing dermal tissues at day 28 following subQ material injection, Figure S16 (Supporting Information) showed that for all material formulations, the injected microparticles consolidated into a compact, macroscopic mass (Figure S16A(i)–D(i), Supporting Information) with several layers of cellular fibrosis at the outer edge of the mass (Figure S16A(ii)–D(ii), Supporting Information). Within the inner region of this mass, discrete microparticles could still be observed with collagen fibres and single‐nucleus fibroblasts infiltrating into the inter‐particle space (Figure S16A(iii)–D(iii), Supporting Information), indicating that the bulk of the injected microparticles were not phagocytosed. We speculate that the aggregation of a large number of microparticles into a compact, macroscopic mass might have reduce the tendency for phagocytosis of discrete microparticles^[^ ^66^, ^67^ ^]^ and instead, induced a fibrotic reaction similar to foreign body response. However, quantitative analysis of fibrosis formation would require more robust data acquisition to avoid sample breakage and rigorous quantification technique to eliminate human bias. Since these techniques are beyond the scope of this study, we refrained from deducing any quantitative relationship between protease activity with fibrosis formation.

### 2.6 Protease Activity as a Predictive Biomarker for Anti‐Inflammatory Effect of Bioactive Compounds in Subcutaneous Material‐Induced Host Response

**Figure 6:**
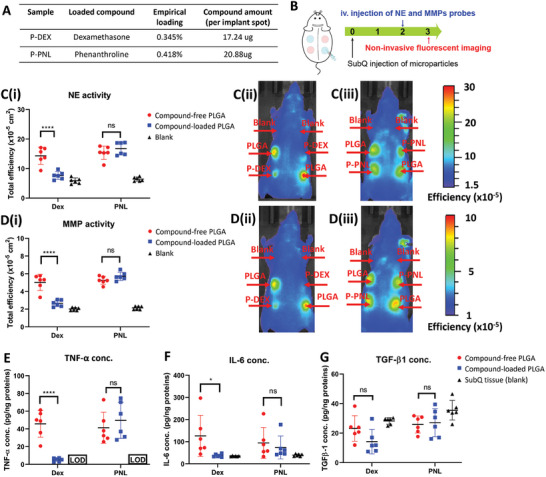
Effect of bioactive compounds loaded in PLGA microparticles on protease activity and protein expression of inflammation‐associated biomarkers in subcutaneous material‐induced host response. A) Drug loading characteristics of PLGA microparticles encapsulating dexamethasone or phenanthroline. B) Schematic of experimental design to evaluate effects of microparticles loaded with each candidate compound by fluorescent imaging. C) Fluorescent signal of NE probe (C(i)) and representative fluorescent images of NE probe signal from mice injected with P‐DEX (C(ii)) and P‐PNL (C(iii)) on day 3. D) Fluorescent signal of MMP probe D(i)) and representative fluorescent images of MMP probe signal from mice injected with P‐DEX D(ii)) and P‐PNL D(iii)) on day 3. E–G) Protein concentration of pro‐inflammatory cytokines (E) TNF‐α, (F) IL‐6, and pro‐fibrotic cytokine (G) TGFβ−1 in retrieved subQ tissue containing blank PLGA microparticles and compound‐loaded PLGA microparticles. TNF‐α protein concentration of control subQ tissue in (E) was lower than limit of detection (LOD). Data represents mean ± S.E.M. of N = 6. *p*‐values of C(i),D(i),E–G) were determined by two‐way ANOVA with Tukey's multiple comparison test. ns, (^*^), (^**^), (^***^), and (^****^) denote non significance, *p* < 0.05, *p* < 0.01, *p* < 0.001, and *p* < 0.0001, respectively.

Incorporating bioactive compounds into implanted materials has been proposed as a strategy to modulate host immune response. We further explored the feasibility of leveraging protease activity as a non‐invasively acquired in vivo parameter to predict the effectiveness of bioactive agents in modulating the inflammatory status of the micro‐environment at the material injection site. Herein, PLGA was selected as the carrier material due to the ease of formulating it into degradable controlled release microparticles containing the bioactive compounds of interest. Each of the two model bioactive, small‐molecule compounds, namely dexamethasone (DEX) and phenanthroline (PNL), was encapsulated in these PLGA microparticles at similar drug loading (≈0.3–0.4%) (**Figure** **6**A) and evaluated for their effects on in vivo NE and MMP protease activity via non‐invasive fluorescent imaging (Figure 6B–D) as well as the protein expression levels of inflammation‐associated markers (Figure 6E–G). Each mouse received 4 subcutaneous injections (Figure S17, Supporting Information) comprising two spots of blank PLGA microparticles and two spots of compound‐loaded PLGA microparticles, which were either dexamethasone‐loaded PLGA microparticles (P‐DEX) or phenanthroline‐loaded PLGA microparticles (P‐PNL).

Figure 6C,D summarizes fluorescent imaging data from both NE and MMP‐activatable probes at the material injection sites, demonstrating that P‐DEX suppressed activities of both proteases while P‐PNL did not affect the activity of either enzyme. Specifically, for each protease, the fluorescent signals at the P‐DEX injection sites were lower than those with compound‐free PLGA microparticles and similar to the levels observed at the blank control sites without material (Figure 6C(i,ii),D(i,ii)). This suppressive effect of dexamethasone on protease activity induced by PLGA was also localized at the P‐DEX injection sites and did not eliminate the signals at adjacent sites injected with compound‐free PLGA microparticles. Conversely, the presence of phenanthroline in P‐PNL did not significantly impact protease activities of both NE (Figure 6C(i,iii)) and MMP (Figure 6D(i,iii)), as the signal from the P‐PNL injection sites was comparable to that from the sites with compound‐free PLGA within the same mouse.

Parallel ex vivo analysis of inflammation‐associated cytokines in retrieved material‐containing subcutaneous tissues (Figure 6E,F) confirmed similar findings to results based on protease activities (Figure 6B,C), demonstrating that P‐DEX suppressed protein expression of pro‐inflammatory cytokines TNF‐α and IL‐6 while P‐PNL had no significant effect on these cytokines. Specifically, P‐DEX decreased the expression of TNF‐α and IL‐6 (Figure 6E,F) to levels close to equivalent values found in blank subQ tissues, which were significantly below the levels induced by compound‐free PLGA microparticles. In contrast, the presence of phenanthroline in P‐PNL had no notable effect on both of these cytokines. Meanwhile, P‐DEX and PNL exerted no significant inhibitory effect on expression of TGF‐β1 in comparison to compound‐free PLGA microparticles (Figure 6G). Taken together, the data in Figure 6 demonstrated that protease activity exhibited a predictive alignment with protein expression of pro‐inflammatory cytokines, corroborating the potential utility of this non‐invasive, image‐based parameter in assessing the anti‐inflammatory effects of bioactive compounds on subcutaneous material‐induced host response.

## 3 Further Discussion

The role of pro‐inflammatory proteases has been elucidated in both physiological and pathological processes such as wound healing, embryo implantation, bone resorption, and metastatic cancers.^[^ ^68^, ^69^, ^70^, ^71^, ^72^ ^]^ Proteolytic activities also play a significant role in the degradation‐elimination of foreign microorganisms and the alteration of the ECM.^[^ ^73^ ^]^ Furthermore, these proteases are also regulators of receptor specificity and activity of cytokines, as well as associated inflammatory cellular processes.^[^ ^11^, ^14^ ^]^ Since material‐induced host response is an immune‐mediated process characterized by inflammation and its resolution in the presence of foreign materials, proteases constitute a group of promising candidate biomarkers for monitoring this host immune responses. This study comprehensively profiled the presence of proteases such as NE, MMPs, Cat‐K, and Cat‐B in material‐induced host response by examining their corresponding mRNA expression, protein expression and activity level to provide new insights on their relevance in material‐induced host response.

Monitoring protease activity by non‐invasive imaging with fluorescent activatable probes has been utilized to evaluate the extent of inflammation in material‐induced host response.^[^ ^20^ ^]^ In this study, protease molecular expression and their activity were analyzed at two distinct time points during the first 9 days after subQ material injection in immuno‐competent mice. Previous studies have established that protease activity is most intense within this period following subQ material injection.^[^ ^20^, ^21^, ^34^ ^]^ Specifically, day 3 post‐implant was herein chosen as the time point to characterize early material‐induced host response as it represents the beginning of the acute inflammation phase. During this acute phase of inflammation characterized by the interplay between neutrophils, monocytes, and macrophages,^[^ ^74^ ^]^ proteases have been postulated to regulate cellular transmigration and recruitment of immune cells to inflamed sites.^[^ ^75^ ^]^ Afterward, the material‐induced host response was assessed at day 9 at the subsequent inflammation stage when the acute response was expected to be dampened.^[^ ^20^, ^21^ ^]^

As the immune cell composition dynamically changes during different stages of inflammation, cellular activities could affect the amount of released or activated proteases. In our study, there was significant divergence in molecular expression levels (mRNA and protein) and temporal activity dynamics of proteases in response to different types of implanted materials. Data in Figures 2 and 4 indicated that the protease activity and protein level of NE and MMPs increased in response to PS microparticles at day 9 compared to that of day 3 following material injection, while the opposite phenomenon was seen with PLGA. In contrast, profiling different subpopulations of immune cells at material‐induced inflammation site does not always produce such distinctive material‐specific signature. For example, Siddharth et. al. reported similar extent of infiltration by multiple immune cell types such as neutrophils, T cells and B cells to the peritoneal cavity following implants of five materials with distinct characteristics, while macrophages and dendritic cells only exhibited elevated responses toward alginate.^[^ ^76^ ^]^ Thus, while the characteristics of cellular reaction and infiltration to implanted materials are important markers to evaluate their immuno‐compatibility,^[^ ^77^, ^78^ ^]^ relying solely on characterization of immune cells might not be sufficiently comprehensive to differentiate the extent of host immune response induced by different types of materials. Hence, a dynamic profile of proteases could synergize with cellular characterization to reflect more comprehensively the progression of material‐induced host response.

We further demonstrated that a comprehensive profile of protease expression from mRNA and protein concentration to protease activity would be required to reliably evaluate the role of proteases in material‐induced host response. Our findings indicated that the molecular concentrations and activity of a protease might exhibit notable deviation from each other (Figures 2 and 4). In fact, they provided complementary information that would be meaningful in comparative analyses of materials‐host response. In subQ tissues containing PLGA and PS‐NH_2_ at day 9 after microparticle injection, significantly different NE protease activities were observed (Figure 2B(ii)) but their NE protein concentrations remained similar (Figure 4A(ii)). In contrast, PS‐NH_2_ and PS microparticles induced different NE and MMP9 protein concentrations of these immune proteases at day 3 (Figure 4A(i),B(i)), but their corresponding protease activities were similar (Figure 2B(i),C(i)).

Proteases are produced by active immune cells^[^ ^76^ ^]^ in their latent forms known as pro‐proteases, which subsequently undergo maturation processes to gain catalytic activity.^[^ ^79^, ^80^ ^]^ However, not all pro‐protease molecules will be converted to their active forms, with some remaining inactive for eventual degradation.^[^ ^81^ ^]^ The discrepancy between protein concentration of proteases (Figure 4) and their activities (Figure 2) could be partially attributed to the existence of proteases at inflammation sites in both their active form and inactive pro‐form, as well as the active form being inhibited by endogenous inhibitors in specific neutrophilic granules.^[^ ^82^ ^]^ These pro‐forms and inhibited proteases could become fully active at a later stage.^[^ ^81^ ^]^ Specifically, the data acquired using protease‐activable imaging probes (Figure 2) only captured the activity of fully processed, mature proteases during the non‐invasive imaging experiments on day 3 or day 9 after material injection. In contrast, the quantitative analysis of protein concentration for each protease (Figure 4) should capture the amount of all protease forms comprising pro‐forms, active forms and inhibited proteases, with each of these forms including both its intracellular and extracellular configurations.^[^ ^83^, ^84^, ^85^ ^]^ Thus, both concentration and activity of inflammatory proteases are partially indicative of the intensity of host immune response. However, it is likely that activity quantification can better reflect the real‐time effect of proteases on the inflammation microenvironment because active proteases are the main contributors in regulating the infiltration of immune cells and modulating signaling molecules in subsequent inflammation cascades.^[^ ^81^, ^86^ ^]^ Hypothetically, a study examining only protein concentration in the case of PS and PS‐NH_2_ at day 3 might result in an inadequate conclusion highlighting the immune‐triggering nature of PS‐NH_2_ microparticles; whereas, in reality, the implant sites of both materials could induce similar inflammation levels at that time point with comparable effects of proteases activities on the destruction of the ECM and immune cell migration.^[^ ^87^ ^]^ Thus, we recommend that future studies should evaluate proteases at both molecular levels and their activities to enable more comprehensive assessment of the extent of the host response against a specific material.

Furthermore, the major role of proteases in regulating cellular infiltration and cytokine expression in multiple inflammation‐associated pathological processes supports the hypothesis that protease activities would also influence the molecular milieu surrounding injected materials. This is because the presence of a foreign, non‐self material disturbs the homeostatic balance of interacting immune components, resulting in an abnormal, disease‐like deviation from the healthy, uninjured physiological state of the surrounding tissues. Notably, this study demonstrated that protease activities of NE, MMP and Cat‐K exhibits strong positive, monotonic correlations (Figure 5D) with protein expression of the key pro‐inflammatory cytokine TNF‐α, whose upregulation is typically associated with pro‐inflammatory state. While this observation of alignment between protease activity and expression of TNF‐α (Figure 5D) remains a correlation and not a causation analysis, this data suggests that protease activity might have potential utility as a non‐invasive, quantitative pro‐inflammatory parameter that is predictive of the inflammation status of the subQ microenvironment at the microparticle injection sites. This causative hypothesis is corroborated by prior studies demonstrating that proteases regulate functions of pro‐inflammatory cytokines TNF‐α and IL‐6 by cleaving these cytokines into smaller and inactive fragments, resulting in suppression of downstream inflammatory process.^[^ ^15^, ^16^ ^]^

As demonstrated in Figure 6, a potential application of evaluating protease activity is to screen for effective anti‐inflammatory formulations of bioactive agents, which can be used to modulate material‐induced subcutaneous host response. Even though prior studies suggest that dexamethasone is a broad‐spectrum inhibitor of inflammation and phenanthroline is a potent MMP‐specific inhibitor, comparison of the anti‐inflammatory effects of these compounds in the context of modulating material‐induced host response was not previously known. Interestingly, the analysis of protease activity herein revealed that, at the similar dosage evaluated, dexamethasone‐loaded P‐DEX exhibited protease‐inhibitory effect while phenanthroline‐loaded P‐PNL surprisingly did not. Importantly, a parallel experiment analyzing cytokine expression in retrieved material‐containing subcutaneous tissues independently resulted in the same conclusion about the inhibitory effects of both compounds. We envision that expanding beyond this initial proof‐of‐concept study with two model compounds, a similar approach could be used to evaluate novel compounds to gain insight into their effects on modulating the immune environment surrounding implanted biomaterials. Such strategy should begin with non‐invasive time‐lapse acquisition of imaging data for protease activity at early time points. Only after lead compounds are identified as inhibitors of protease activity should subsequent tissue retrieval be conducted to obtain more comprehensive molecular and cellular data and confirm the findings.

In addition, to accurately quantify the protease activities using non‐invasive imaging, careful evaluation and selection of suitable biomarkers and imaging probes are required. As demonstrated herein, the subcutaneous mouse model proved to be suitable for monitoring host immune response against foreign materials using MMPs, NE, and Cat‐K as biomarkers. However, the probe for Cat‐B was shown to be an unexpected exception among the chosen imaging agents. Specifically, ex vivo analysis proved that Cat‐B protease was present in the microparticle‐containing subQ tissues retrieved from mice, inducing increase in fluorescent signal after 24‐h incubation with its corresponding imaging probe (Figure S3D, Supporting Information). However, a similar increase in Cat‐B‐activated fluorescent signal was not observed in vivo, where the signal did not increase but quickly faded immediately after probe injection (Figure 1F; Figure S9, Supporting Information). Given that Cat‐B is present at the retrieved target material‐containing tissue as demonstrated in the *ex vivo* experiment, this in vivo phenomenon might be due to in vivo distribution, accumulation, and clearance kinetics of the Cat‐B imaging probe at the subQ space. The opposite scenario was observed with Cat‐K as its in vivo fluorescent signals are fully detectable (Figure 1E) while their protein level fell under the detection limit of our chosen ELISA assay. Our mRNA analyses (Figure 5) corroborated the findings with in vivo fluorescent imaging of Cat‐K activity (Figure 2), demonstrating that Cat‐K was present at both examined time points and increased at day 9 post‐microparticles injection. However, the undetectable Cat‐K protein level with ELISA could be the basis for speculation that a small amount of protease Cat‐K could still interact with the administered probes and gave out measurable fluorescent signals. The upregulation of mRNA expression for Cat‐K on day 9 compared to that on day 3 also suggests that Cat‐K may be suitable for longitudinal studies of material‐induced host response over a longer time frame. Cat‐K protease was previously found to play critical role in regulating the dermal ECM, particularly its matrix synthesis and degradation in scar formation after 1 month.^[^ ^88^ ^]^ Thus, in this subcutaneous material‐induced host response model, we speculate that Cat‐K proteases could also be more involved and upregulated in later time points where wound healing occurs, rather than the early time of acute inflammation response.

Another key insight gained from this study indicates that the in vivo distribution and clearance kinetics of the probes for the specific animal model of interest needs to be assessed once suitable biomarkers and imaging probes have been identified for an intended application. After probe injection, the optimal time points for data acquisition varies markedly between different animal models and the target tissue types.^[^ ^89^, ^90^ ^]^ Typically, after intravenous injection, the administered probe enters systemic circulation and is subsequently distributed to the material‐injected subcutaneous site under the skin. At the material‐injected sites, the probe is cleaved and activated by its corresponding protease to emit fluorescence when imaged. Concurrently, probe is also cleared via subcutaneous vasculature to the main circulation and eventually excretory organs, thus causing a simultaneous drop in probe concentration at the material‐injection sites. A dynamic process exists at the material‐injection site involving probe arrival from and clearance to systemic circulation as well as probe activation by the active protease present. Over the initial time period immediately after probe injection, the concentration of activated probe and thus fluorescent signal increase gradually till the signal peaks, indicating maximum probe activation possible by the active protease available at the material‐injected sites before probe clearance becomes predominant. In this study, this dynamics of probe distribution, activation and clearance was captured in Figure 1C–E, which showed that the graph for distribution and clearance kinetics for each imaging probe was of the same shape with the same time point of peak fluorescence regardless of the materials injected. Qualitatively, the shape of each distribution/clearance graph and its corresponding peak time only changed with different types of imaging probes, suggesting that the characteristics of the probe molecule, and not the material formulation, was the primary factor affecting the distribution/clearance kinetics. For each graph, only the peak height varied with different types of material formulations and since Figure S8A,B (Supporting Information) proved that there was no differential probe‐material adsorption, the peak height variation must have primarily resulted from the differential protease activity. Interestingly, in this study involving assessment of implanted materials in subcutaneous space of SKH1E mice, the signals corresponding to the activity of the chosen proteases peaked at a different time point from the manufacturer's recommendation (Figure 1). For instance, NE probes were recommended for imaging 4–8 h post‐injection in acute lung injury model on CD‐1 female mice (Charles River).^[^ ^54^, ^91^ ^]^ For tumor graft in BALB/C mice, the manufacturer recommended to image 24 h after the injection^[^ ^56^, ^92^ ^]^ whereas this duration is 6 h for Cat‐K probes in BALB/c mice arthritis model.^[^ ^55^, ^93^ ^]^ From our investigation, the peak time for maximum fluorescent signal in the mouse subcutaneous model was 24 h for NE, MMPs, and 9 h for Cat‐K. This time point of peak signal was selected for image acquisition to achieve the highest signal‐to‐noise ratio, which was the ratio of material‐induced signal to signal from control blank skin, and maximize the resolution or difference in the signals induced by different types of materials. Additionally, to eliminate the effect of injection position on probe distribution kinetics, the experiments herein were designed such that the injection positions of each microparticle formulation were alternated over all four possible positions on the dorsal side of each mouse across N = 10–12 biological repeats as shown in the injection maps for Figures S2 and S17 (Supporting Information). The reported fluorescent signal for each microparticle formulation was averaged from multiple different injection positions hence eliminating site‐dependent delay, if any, in the clearance of measured signal. Notably, the averaged signal has small standard deviation, confirming that position of injection site has minimal impact on the data in our experimental design. The fluorescent signal on day 6 following i.v injection of probes (Figure 1C,D) suggests that there might be a small residual amount remaining uncleared. Nonetheless, the variation in the residual amount across materials was minimal and still likely originated from the differential material‐dependent protease activity, similar to the differential signal at peak time and not due to adsorption‐dependent or site‐dependent delayed clearance.

Besides these considerations, another unique issue in utilizing probe‐based imaging methods for materials‐host immune response is the possibility of artifact signal caused by the materials. Dang et al. previously detected the autofluorescence of implanted PLGA microparticles and used its signal as a marker of cellular coverage on the particles.^[^ ^34^ ^]^ As shown in our in vitro assessment (Figure S6, Supporting Information), microparticles interacting with probes could also generate false signals without the presence of the targeted proteases. Similar activatable probes for non‐invasive imaging have been utilized for evaluation of material‐induced immune response,^[^ ^20^, ^21^, ^36^ ^]^ but this artifact interaction had not been considered or reported in the literature. Nonetheless, this artifact was not observed when the particles were covered in subQ tissues or serum proteins such as albumin (Figures S3 and S7, Supporting Information). However, the mechanism of this artifact and its suppression is unclear, so this issue should be carefully considered in future development and selection of fluorescent imaging probes for application involving implanted materials. We speculate that this artifact enhancement in the fluorescent signal might have resulted from hydrophobic interaction between PS or PS‐NH2 microparticles with the fluorochrome moieties on each imaging probe in the absence of serum proteins. Each imaging probe consist of multiple flourochromes conjugated to a polymeric backbone of PEGlytated poly‐L‐lysine.^[^ ^85^ ^]^ Prior to probe activation by protease activity, inactivated flourochromes in close proximity exhibits self‐quenching, possibly due to formation of an intramolecular dimer between reporter and quencher, to create a non‐fluorescent ground‐state complex. Upon addition of protein‐free PS or PS‐NH2 microparticles, the hydrophobic aromatic rings of the fluochrome moieties might interact with similar aromatic benzyl groups of the polystyrene backbone. This hydrophobic interaction possibly results in disruption of the stable complex to free the fluorochromes and thus increasing its fluorescent signal. If serum proteins or BSA were added, the protein corona adsorbed on the PS or PS‐NH_2_ microparticles would shield the polystyrene surface from direct contact with the imaging probe, hence preventing any interaction between the fluorochromes and polystyrene from destabilizing the self‐quenching of the probe. Thus, necessary pre‐screening of probe‐material interactions as we presented herein (Figures S3,S6,S7, Supporting Information) would need to be conducted prior to in vivo evaluation to ensure the reliability of the method for monitoring of protease activity.

Arguably, in addition to chemical composition of a material formulation, multiple physical variables such as shapes of particles, amount injected, material porosity can also contribute to the protease activity measured. Strictly speaking, each “material” mentioned herein refers to a “material formulation” resultant from the combined characteristics of all physico‐chemical parameters of the microparticles used. The different microparticle formulations used in our study were chosen mainly to allow us to investigate a range of different protease response and the differential response measured would, in principle, be attributed to the combinatorial effects of all variables for each formulation and not to its chemical composition alone. Nonetheless, to standardize some of these variables, we designed the experiment such that the amount of particles injected for each material formulation is consistent (5 mg per injection); the shape of particles for each formulation was spherical (Figure S1, Supporting Information) and the size of the microparticles was within the diameter range of 5–7um(Figure S1, Supporting Information). Therefore, the data reported for each material formulation can still be primarily attributed to material chemical composition and its derivative parameters such as density and hydrophobicity/hydrophilicity, which are inherently dependent on how the chemical composition influences the characteristics of the final material formulation. Additionally, the host response evoked by our injected microparticles might not completely capture the phenomenon of “foreign body response” as how this term was strictly defined in classical biomaterial literature.^[^ ^6^, ^94^ ^]^ However, Figure S16 (Supporting Information) revealed that for all material formulations, the injected microparticles consolidate into a compact, macroscopic mass with several layers of cellular fibrosis at the outer edge of the mass (Figure S16A(ii)–D(ii), Supporting Information). The compaction of the injected microparticles into a macroscopic mass and the observed formation of fibrotic cellular layers around this macroscopic mass are important indicators that the host response we observed did exhibit major characteristics of a host response against a macroscopic foreign body of the intended materials. “Breaking down” a material into microparticle format to allow it to be injected into the subcutaneous space reduced potential confounding effect of additional injuries required by administration of larger material blocks. The in vivo re‐aggregation of the microparticle back into a compact large mass following microparticle injection allows this system to retain essential characteristics of the FBR such as fibrotic cellular layers surrounding the material mass while reducing the vulnerability to phagocytosis of discrete microparticles. This host response to each material system in this study exhibited intermediate characteristics between the response induced by a system of discrete microparticles and that by a large implant of solid mass.

Overall, our comprehensive evaluation of different protease subclasses and a range of materials demonstrated that selected proteases can be useful biomolecular signatures for dynamic profiling of material‐induced host response. The difference in host immune response against different materials is reflected in both the molecular expression levels and the temporal variation of protease activity. Our findings here expanded the limited observation reported in previous literature which have examined only the activity of a few proteases in material‐induced host response.^[^ ^20^, ^21^ ^]^ Considering the major impact of proteases in the material‐induced host response, the new insights reported herein on the dynamic evolution of proteases, such as NE and MMPs, would be useful in establishing them as a characterization tool for material‐induced immune response as well as a therapeutic target. However, the current study cannot provide a more detailed timeline of the protease activity since accurate imaging at each time point requires that the probe from previous instance of administration has been cleared from the animal. This clearance duration could be up to 6 days for NE imaging probe in our mouse model (Figure 1). Thus, the development of substrate‐based imaging probe for protease with faster clearance time while maintaining its specificity would be of high value. This potential advance would enable more comprehensive dynamic profiling of protease activity. Furthermore, besides the proteases demonstrated in this study, other proteases could also be prospective biomarkers for the evaluation of material‐induced host response or future applications involving proteolytic activities. Thus, it would be worthwhile for future investigation to establish an atlas of protease transcriptome and proteome, which could reveal the whole picture on proteases and their dynamic change in material‐induced host response. We envision that such a complete atlas would facilitate future research and application leveraging the role of proteases in material‐induced host response. Specifically, it would assist the design of biomaterials with specific immune‐modulating characteristics such as protease‐triggered drug releases systems or immuno‐modulatory materials.^[^ ^95^ ^]^

## 4 Conclusion

This study reported the dynamic profiling from molecular expression to activity levels of inflammatory proteases (NE, MMPs, Cat‐K, and Cat‐B) in a subcutaneous biomaterial implant model. From these profiles, we demonstrated that proteases are viable biomarkers for evaluation of material‐induced host response. The mRNA, protein expression and protease activities of the chosen proteases differed depending on the type of materials, reflecting different extents of immune response. Likewise, the temporal changes of proteases at these levels were differentially affected depending on the type of materials. However, we highlighted that quantitative protease activities are complementary but not always directly correlated to their molecular expression level, suggesting that a comprehensive protease profile is needed to better assess the level of immune response against implanted materials. We also demonstrated that protease activity exhibited a strong positive correlation with pro‐inflammatory marker TNF‐α and illustrating the predictive utility of this non‐invasive, imaging‐based parameter in assess anti‐inflammatory effects of bioactive compounds for modulation of material‐induced host response. Our findings provided a comprehensive evaluation of proteases in material‐induced host response and potentially benefit future research leveraging the activity of proteases such as material evaluation for medical implants, vaccine adjuvants or protease‐responsive drug delivery systems.

## 5 Experimental Section

### Materials

The following materials were purchased from commercial sources including polystyrene (PS) microparticles of 5 µm diameter (Spherotech, USA), polystyrene microparticles with terminal amino groups (PS‐NH_2_) of 5 µm diameter (Spherotech, USA), Silica (SiO_2_) microparticles of 7.75 µm diameter (Cospheric, USA), Poly(DL‐lactide‐co‐glycolide) (PLGA) with a 50:50 monomer ratio, and inherent viscosity range of 0.95–1.2 dL g^−1^ (Durect Corporation, Lactel Polymer, Pelham, AL, USA), and polyvinyl alcohol (PVA) (9000–10,000 Da, Sigma Aldrich).

### Formulation of PLGA Microparticles

PLGA microparticles were prepared using a co‐solvent evaporation method as reported in the previous publication.^[^ ^34^ ^]^ Typically, a volume of 5 ml of 4% PLGA (w/v) in dichloromethane was added to a 25 mL solution of 1% PVA (w/v) and homogenized for 60 s at 5000 rpm (Silverson L5, Silverson Machines Ltd, England). For formulation of compound‐loaded PLGA microparticles, either 40 mg of phenanthroline (131 377, Sigma–Aldrich) or 10 mg of dexamethasone (D1961, TCI) was dissolved in dichloromethane along with PLGA to formulate phenanthroline‐loaded PLGA (P‐PNL) or dexamethasone‐loaded PLGA (P‐DEX), respectively. The resulting suspension was decanted into 75 mL deionized water followed by rotary evaporation (Buchi Rotavapor R‐200, Buchi, Switzerland) for removal of dichloromethane. The suspension was washed 3 times with deionized water, followed by collection after three days of lyophilization and subsequent storage at −20 °C for characterization and downstream applications. The morphology of microparticles was examined by scanning electron microscope (JSM 6390LA, JEOL) at 10 keV. Before scanning, the microparticles were deposited onto silicon wafers and sputter‐coated with platinum at 20 mA for 120 s.

### Animal Care

All animal experiments conducted in this study adhered to the guidelines of the National Advisory Committee for the Laboratory Animal Research (NACLAR) and complied with the National Institutes of Health guide for the care and use of laboratory animals (NIH Publications No. 8023, revised in 1978). The animal protocol A19056 was approved by the Institutional Animal Care and Use Committee (IACUC) of Nanyang Technological University (NTU), Singapore prior to the initiation of the study. This study used SKH1‐E mice aged between 10 and 15 weeks (F1) bred in‐house using breeder hairless immunocompetent SKH1‐E mice (strain code 477)^[^ ^96^ ^]^ purchased from Charles River Laboratories (Wilmington, MA, USA). The mice were housed under standard conditions with a 12‐h light/dark cycle at Research Support Building of Lee Kong Chian School of Medicine (NTU, Singapore). Both water and food were provided ad libitum.

### Subcutaneous Injection of Microparticles in SKH1‐E Mice

Hairless immuno‐competent SKH1‐E mice were anesthetized by inhalation of 3% isoflurane in oxygen throughout the injection process. The dorsal region of each mouse was disinfected with alcohol prior to injection. Each mouse received four injections of different formulations (PS, PS‐NH2, PLGA, and SiO2) via 23G needle followed in an array format on the dorsal side (Figure 1A). Each formulation contained 100 µL suspension of 50 mg microparticles in 1 mL DPBS (HyClone). For experiments with compound loaded‐PLGA microparticles, each mouse received 4 subcutaneous injections comprising two spots of blank PLGA microparticles and two spots of compound‐loaded PLGA microparticles, which were either dexamethasone‐loaded PLGA microparticles (P‐DEX) or phenanthroline‐loaded PLGA microparticles (P‐PNL). For each set of experiments, the injection positions of each microparticle formulation were alternated over all four positions possible on dorsal side of each mouse across N = 10–12 biological repeats, as shown in Figures S2 and S17 (Supporting Information).

### Non‐Invasive Fluorescent Imaging of SKH1‐E Mice

SKH1‐E mice were strictly fed with a non‐fluorescent alfalfa‐free diet (Teklad Irradiated Rodent Diet, Madison, WI, USA) starting from two weeks prior to microparticle injection until the end of the experiment. The activity of proteases namely neutrophil elastase (NE), matrix metalloproteinases (MMPs), cathepsin K (Cat‐K) and B (Cat‐B) was quantified based on the fluorescent signal generated by protease‐activatable fluorescent probes. Imaging probes NE (NEV11169, Perkin Elmer), MMPs (NEV10168, Perkin Elmer), Cat‐K (c, Perkin Elmer) and Cat‐B (NEV11098, Perkin Elmer) were dissolved in sterile PBS prior to intravenous injection with the recommended doses from the manufacturer. The excitation and emission wavelengths of NE and Cat‐K were 675 and 720 nm respectively and those of MMPs and Cat‐B were 745 and 800 nm, respectively. Since there are two different windows of excitation and emission wavelengths, each mouse received simultaneously a pair of NE^[^ ^54^ ^]^ and MMPs^[^ ^56^ ^]^ probes or a pair of Cat‐K^[^ ^55^ ^]^ and Cat‐B^[^ ^57^ ^]^ probes. The use of imaging probes in pair was performed according to the manufacturer's recommendations to minimize signal interference between the probes of each pair.^[^ ^97^ ^]^ Previous publications and reported spectra of selected probes (Figure S18, Supporting Information) have also demonstrated negligible overlap in excitation/emission between the simultaneously injected probes of each pair.^[^ ^89^, ^91^, ^92^, ^93^, ^98^, ^99^ ^]^ The fluorescent intensity generated from probes after injection was recorded by IVIS spectrum CT system (Perkin Elmer, MA, USA). Data analysis based on the region of interest (ROIs) was performed using the manufacturer's software Living Image 4.7. ROIs were determined around the injection sites for calculation of fluorescent efficiency (cm^2^), which is the ratio of fluorescent intensity between the emission signal to the incident excitation signal.

### Quantification of Protein Expression

Mice were euthanized by CO_2_ asphyxiation on the day of tissue extraction. The dorsal skin containing spots of injected microparticles was harvested and fixed on a styrofoam pad (Figure S5A, Supporting Information). Each of the subQ tissue layer containing the microparticle spots were detached from the dermal layer with a scalpel and put in a tube of 500 µL cold buffer containing 150 mM NaCl, 1% Triton X‐100, 1 mM EDTA, and a protease inhibitor cocktail at a ratio of 1:200 (535 140, Calbiochem) in 10 mM Tris buffer (pH 7.4) (Figure S5B, Supporting Information). A subcutaneous tissue layer without the epidermis and dermis was also harvested from a region without injected microparticles as a control sample (SubQ tissue, Figure S10B, Supporting Information). The collected samples were homogenized using TissueLyser LT (Qiagen, UK) with two stainless steel beads (mean diameter of 5 mm) for 5 min at 50 Hz, followed by 10 min of centrifugation at 10000 rcf at 4 °C to remove insoluble content. The protein concentration of the supernatant was quantified using BCA (23 225, Thermo Scientific). For each sample, a total protein mass of 0.5, 10, and 1 µg was then analyzed to determine the normalized protein expression of Neutrophil elastase, MMP3 (DY548, RnDSystems) and MMP9 (DY6718, RnDSystems) respectively by enzyme‐linked immunosorbent assELISA). Similarly, a total protein mass of 100 µg for each sample was also analyzed with ELISA to determine the normalized protein expression of TNF‐α (430 904, BioLegend), IL‐6 (DY406‐05, RnDSystems), and active TGF‐β1 (DY1679‐05, RnDSystems)

### Quantification of mRNA Expression

For this experiment, tissues extracted were stored immediately in RNAlater solution before being lysed with TRIzol reagent (Invitrogen) according to the manufacturer's instructions. In addition, to ensure complete tissue disruption, the tissues were further minced and lysed by a TissueLyser LT homogenizer (Qiagen, US). Before reverse transcription using M‐MLV Reverse Transcriptase (M1701, Promega), all samples were normalized for comparison by loading the same amount of 1 µg total RNA in a volume of 20 µl. The resulting cDNA (9 µl; 1:6 dilution) was amplified by qPCR using StepOnePlus (Applied BioSystem) with 1 µl of the specific primer (Table S2, Supporting Information) by using iTaq Univeral SYBR Green supermix (1 725 121, Bio‐Rad Laboratories, Inc. California, USA). The amplification process comprised 5 min at 95 °C for enzyme activation, followed by 40 amplification cycles each consisting of 10 s at 95 °C, 10 s at 55 °C and 20 s at 72 °C.

### Statistical Analysis

All data were statistically analyzed and graphed with GraphPad Prism 7.0 software. Most experiment data were expressed as the mean ± S.D or the mean ± S.E.M. for in vitro or in vivo studies, respectively, Comparison of multiple groups was made using one‐way ANOVA analysis followed by Tukey test, while the comparison of a same group between day 3 and day 9 was determined by paired 2 tailed *t*‐test. For correlation analysis between nine biological variables including protease activity (NE‐activity, MMP‐activity, CatK‐activity), protease protein expression (NE conc., MMP9 conc., MMP3 conc.) and inflammatory cytokine protein expression (TNF‐α conc., IL‐6 conc., TGFβ−1 conc.) (Figure 6), a Spearman correlation coefficient was calculated for each pair of these variables using 10 sets of retrieved subQ tissues with or without injected materials at day 3 or day 9. The data for each set of retrieved subQ tissues was the average of N = 9–10 injection repeats. For fold change of mRNA expression of specific MMP proteases (Figure 3), the data were expressed as medians ± IQR (interquartile range) and the statistical difference was determined by non‐parametric test (Kruskal–Wallis test) followed by multiple comparison Dunn's test. For Figure 3 and Figure S14 (Supporting Information), outliers were excluded by performing Grubb's outlier tests. For all comparative analysis, *p*<0.05 was considered significant. Significant difference was expressed as *p < 0.05, **p < 0.01, ***p < 0.001 and ****p < 0.0001.

## Conflict of Interest

The authors declare no conflict of interest.

## Supporting information



Supporting Information

## Data Availability

The data that support the findings of this study are available from the corresponding author upon reasonable request.
