# Supplementary material for: Profiling Pro‐Inflammatory Proteases as Biomolecular Signatures of Material‐Induced Subcutaneous Host Response in Immuno‐Competent Mice
Source: Adv Sci (Weinh). 2024 Dec 4;12(5):2309709. doi: 10.1002/advs.202309709 (PMC11792001; doi:10.1002/advs.202309709)
Supplement: Supplementary file 1 — Supporting Information [file ADVS-12-2309709-s001.docx]

**Profiling Pro-inflammatory Proteases as Biomolecular Signatures of Material-induced Subcutaneous Host Response in Immuno-competent Mice**

Nam M.P. Tran^#^, Anh T.H. Truong^#^, Dang T. Nguyen, Tram T. Dang*

School of Chemistry, Chemical Engineering, and Biotechnology, Nanyang Technological University, 70 Nanyang Drive, Singapore 637459, Singapore

^#^ These authors contribute equally to this work

* All correspondence to be addressed to ttdang@ntu.edu.sg

**SUPPLEMENTARY INFORMATION**

## TABLES

**Table S1. Abbreviation**

| BSA | Bovine serum albumin |
| --- | --- |
| Cat-B | Cathepsin B |
| Cat-K | Cathepsin K |
| DEX | Dexamethasone |
| ECM | Extracellular matrix |
| ELISA | Enzyme-linked immunosorbent assay |
| FACS | Fluorescence-activated cell sorting |
| GAPDH | Glyceraldehyde 3-phosphate dehydrogenase |
| IL-2 | Interleukin-2 |
| IL-4 | Interleukin-4 |
| IL-6 | Interleukin-6 |
| IL-8 | Interleukin-8 |
| IL-13 | Interleukin-13 |
| IQR | Interquartile range |
| MMPs | Matrix metallo-proteinases |
| NE | Neutrophil elastase |
| PBS | Phosphate-buffered saline |
| P-DEX | Dexamethasone-loaded PLGA microparticles |
| PLGA | Poly(lactic-co-glycolic acid) |
| PNL | Phenanthroline |
| P-PNL | Phenanthroline-loaded PLGA microparticles |
| PS | Polystyrene |
| PS-NH_2_ | Polystyrene with terminal amino groups on the surface |
| SiO_2_ | Silica |
| subQ | subcutaneous |
| RT-qPCR | Real-time quantitative Polymerase chain reaction |
| TGF-β | Transforming growth factor-β |
| TNF-α | Tumor necrosis factor alpha |

**Table S2. Primer sequences utilized for qPCR performance (designed by PrimerQuest™ Tool from IDT)**

| **Gene** | **Forward primer**  **(5’**→**3’)** | **Reverse primer**  **(5’**→**3’)** |
| --- | --- | --- |
| **GAPDH** | CCACTGGTGCTGCCAAGGCT | GGCAGGTTTCTCCAGGCGGC |
| **MMP-2** | CAAGTTCCCCGGCGATGTC | TTCTGGTCAAGGTCACCTGTC |
| **MMP-3** | ACATGGAGACTTTGTCCCTTTTG | TTGGCTGAGTGGTAGAGTCCC |
| **MMP-7** | TTGCAGGCATTCAGAAGTTATATGG | GTTTGTTCATGCCAGCTGAGG |
| **MMP-9** | GTGTTCCCGTTCATCTTTGAGG | GGCAGAAGCCATACAGTTTATCC |
| **MMP-12** | GAGTCCAGCCACCAACATTAC | GCGAAGTGGGTCAAAGACAG |
| **MMP-13** | AGTAGATCCAGCTAAGACACAGC | AAACATGGTGGAGCACAAAGG |
| **Cat-K** | CAGTAGCCACGCTTCCTATCC | GTAGCAGCAGAAACTTGAACACC |
| **NE** | GCCTAAATTTCCGGTCAGTGC | CAGTTGTGATGGGTCAAAGCC |

**Table S3. Experimental replication to acquire the standard-errors-of-the-mean (S.E.M).**

| **Figure** | **Replication** |
| --- | --- |
| **1** | For the *in vivo* experiment demonstrating distribution and clearance kinetics of fluorescent probes, a pair of NE and MMPs probes were simultaneously injected intravenously via tail veins into N=10 SKH1-E mice, while the pair of Cat-K and Cat-B probes were similarly administered in another N=9 mice. Four different material formulations were subcutaneously injected into 4 separate spots on the dorsal side of each mouse. The injection positions of each microparticle formulation were alternated over all four positions possible on dorsal side of each mouse across N=10 biological repeats, as shown in **Figure S2**. |
| **2** | The fluorescent signals indicating protease activity were quantified based on the fluorescent intensity within the regions of interest (ROIs) around the microparticle injection spots on the same mice used for **Figure 1**, specifically N=10 mice for the pair of NE and MMPs probes, and N=9 mice for Cat-K probe. |
| **3** | For the evaluation of mRNA expression of specific MMP proteases, 4 material formulations were subcutaneously injected onto the dorsal side of each mouse. The injection positions of each microparticle formulation were alternated over all four positions possible on dorsal side of each mouse across N=10 biological repeats, similar to the one shown in **Figure S2**. Ten mice were injected with microparticles for each time point of day 3 or day 9. Each retrieved microparticle-containing subcutaneous tissue was separated into 2 equal portions. One portion was used for mRNA analysis in this experiment for **Figure 3** while the other portion was used for protein analysis in **Figure 4**. N=10 samples were used in data analysis for each material at each time point, except for PS-Day 3 and SiO_2_-Day 9 with N=9. One data point from the retrieved tissue containing injected PS microparticles on day 3 (PS-Day 3) was removed because the Grubb’s outlier test identified it as an outlier (p < 0.0001). One of the samples of retrieved tissue containing injected SiO_2_ on day 9 (SiO_2_-Day 9) was lost by mistake during sample processing for mRNA analysis. |
| **4** | For the quantification of total protease protein from retrieved subcutaneous tissues containing injected materials, 4 material formulations were subcutaneously injected onto the dorsal side of each mouse. The injection positions of each microparticle formulation were alternated over all four positions possible on dorsal side of each mouse across N=12 biological repeats, similar to the one shown in **Figure S2**. Ten mice were injected with microparticles for each time point of day 3 or day 9. Each retrieved microparticle-containing subcutaneous tissue were separated into 2 equal portions. One portion was used for protein analysis in this experiment for **Figure 4** while the other portion was used for mRNA analysis in **Figure 3**. The same lysate from each retrieved tissue was used to quantify the protein amount of NE, MMP9, and MMP3. |
| **5** | The quantification of protein expression of pro- and anti-inflammatory cytokines from retrieved subcutaneous tissues containing injected materials was performed on the same lysate samples from the same mice used for **Figure 4.**  For correlation analysis between 9 biological variables including protease activity (NE-activity, MMP-activity, CatK-activity), protease protein expression (NE conc., MMP9 conc., MMP3 conc.) and inflammatory cytokine protein expression (TNF-α conc., IL-6 conc., TGFβ-1 conc.) (**Figure 5**), a Spearman rank-order correlation coefficient was calculated for each pair of these variables.  All variables have a sample size of 10, obtained from 10 sets of retrieved subQ tissues with or without injected materials (PS, PS-NH_2_, PLGA, SiO_2_ and blank/control tissues) at two timepoints, day 3 or day 9. For each variable, the data for each set of retrieved subQ tissues was the average of N=9-10 injection repeats. |
| **6** | For the *in vivo* experiment examining protease activity of NE and MMP, each mouse received 4 subcutaneous injections comprising two spots of blank PLGA microparticles and two spots of compound-loaded PLGA microparticles, which were either dexamethasone-loaded PLGA microparticles (P-DEX) or phenanthroline-loaded PLGA microparticles (P-PNL). The injection positions of each microparticle formulation were alternated over all four positions possible on dorsal side of each mouse across N=12 biological repeats, as shown in **Figure S17.** For the *in vivo* experiment examining protein expression of cytokines, same experimental design was performed with a different set of 12 mice. |

## SUPPLEMENTARY MATERIALS AND METHODS

### *2.1 In vitro interaction of imaging probes with microparticles*

MMP and NE imaging agents (also termed “probes”) were reconstituted in a buffer solution containing 10 mM CaCl_2_, 50 mM HEPES at pH 7.4 (buffer M), while Cat-K and Cat-B probes were reconstituted in another buffer solution containing 50 mM sodium acetate, 1 mM EDTA, 5 mM Cysteine, pH 5.5 (buffer B). To investigate the interaction of imaging probes with microparticles, 5 mg of PS, PS-NH_2_, PLGA or SiO_2_ microparticles was resuspended in 100 µL of buffer M containing 750 nM MMP or 750 nM NE probes, or in 100 µL of buffer B containing 1 µM Cat-K or 750 nM Cat-B probes. To evaluate the suppression of fluorescent signal artifact by BSA, 5% BSA in the corresponding buffer was added into the same mixture of 5 mg PS or PS-NH_2_ microparticles containing imaging probes. Each sample was placed in one well of a 96-well black plate (Corning, USA) with clear bottom. The fluorescent signal of each sample were acquired with IVIS spectrum CT system. Regions of interest (ROIs) were determined with Living Image Software (version 4.7) around the well of 96-well plate for calculation of fluorescent efficiency (cm^2^).

### *2.2 Activation of imaging probes with corresponding proteases in vitro*

To evaluate the activation of each imaging probe with its corresponding protease, 0.1 µg of protease MMP9 (Merck, PF024) or 0.1 µg of NE (Merck, 324681) was added in to 100 µL buffer M with 2 µM of MMP probe or 4 µM of NE probe, respectively. Similarly, 0.04 µg of Cat-K (Merck, 219461) or 0.1 µg of Cat-B (Merck, 219364) was added to 100 µL of buffer B containing 2 µM of Cat-K probe or 4 µM of Cat-B probe, respectively. Each sample was then placed in one well in a 96-well black plate with clear bottom and the fluorescent signals at different time-points were acquired by IVIS spectrum CT system. Regions of interest (ROIs) were determined around the well of 96-well plate for calculation of fluorescent efficiency (cm^2^). Between image acquisition at the 0-hour and 24-hour time points, all samples were incubated at 37°C. To evaluate the *in vitro* activation of each imaging probe with different concentrations of its corresponding protease, similar experiment was performed by adding human recombinant protease MMP9 (Merck, PF024) or human native NE (Merck, 324681) was added into 100 µL buffer M with 750 nM of MMP probe or 750nM of NE probe, respectively.

### *2.3 Activation of imaging probes with retrieved tissues ex vivo*

Similar experiment setting as described in SI section 2.2 was performed to determine the activation of imaging probes but by proteases present in retrieved tissues instead of solution of commercially available proteases. Each retrieved tissue contained 5 mg PS or PS-NH_2_ microparticles that were subcutaneously injected into the dorsal region of each mouse (Figure S11-A). These tissues were retrieved 3 days after injection and immediately added into 100 µL of buffer M with 0.2 nmol of MMP probe or 0.4 nmol of NE probe, or into 100 µL of buffer B containing 0.2 nmol of Cat-K probe or 0.4 nmol of Cat-B probe. Each sample was then placed in one well in a 96-well black plate with clear bottom and the fluorescent signals at different time-points were acquired by IVIS spectrum CT system. Regions of interest (ROIs) were determined with Living Image Software (version 4.7) around the well of 96-well plate for calculation of fluorescent efficiency (cm^2^). Between image acquisition at the 0-hour and 24-hour time points, all samples were incubated at 37°C.

### *2.4 In vitro evaluation of imaging probe adsorption by microparticles*

To evaluate the adsorption of imaging probes by microparticles, 7.5 mg of PS, PS-NH2, PLGA, or SiO2 microparticles were resuspended in either 150 µL of Buffer M with 5% BSA or 150 µL of Buffer B with 5% BSA. Each suspension was incubated in 0.6 mL microcentrifuge tubes at 4°C for 4 hours. After incubation, for samples in Buffer M, MMP or NE imaging probe solutions in PBS were added to achieve a final probe concentration of 750 nM. Similarly, for samples in Buffer B, CatK or CatB imaging probe solutions in PBS were added to a final concentration of 750 nM. Probe solutions mixed in their corresponding buffer containing 5% BSA without microparticles were prepared as control sample. Each sample was then incubated for 18h at 4°C in the dark.

Subsequently, each sample was centrifuged at 10,000 rcf for 10 minutes to allow microparticles to settle. Following centrifugation, 100 µL of the supernatant was transferred to individual wells in a 96-well, black clear-bottom plate. To each well, the protease corresponding to the probe (0.1 µg of MMP9, NE, or CatB, or 0.04 µg of CatK) was added. Fluorescent signals were immediately acquired and after 24 hours using an IVIS Spectrum CT system. ROIs were defined around each well in the 96-well plate to calculate fluorescent efficiency (cm2). Between image acquisitions at 0-hour and 24-hour time points, all samples were incubated at 37°C.

### *2.5 Sample retrieval and histological analysis*

On day 28th after microparticle injection, mice were euthanized via CO2 asphyxiation. Full-thickness dermal and subcutaneous tissues containing the injected microparticles were excised from euthanized mice and fixed overnight in 10% formalin (VWR Chemicals BDH Prolabo). After fixation, the tissues were rinsed thrice with 70% ethanol and stored in 70% ethanol until further processing. Histological analysis was conducted by the Advanced Molecular Pathology Laboratory (AMPL) at the Institute of Cell and Molecular Biology (IMCB, A*STAR, Singapore). Specifically, the fixed tissues and particles were paraffin-embedded in paraffin and processed into sections with thickness of 5 µm prior to their staining with hematoxylin and eosin (H&E) (1).

## SUPPLEMENTARY FIGURES

| 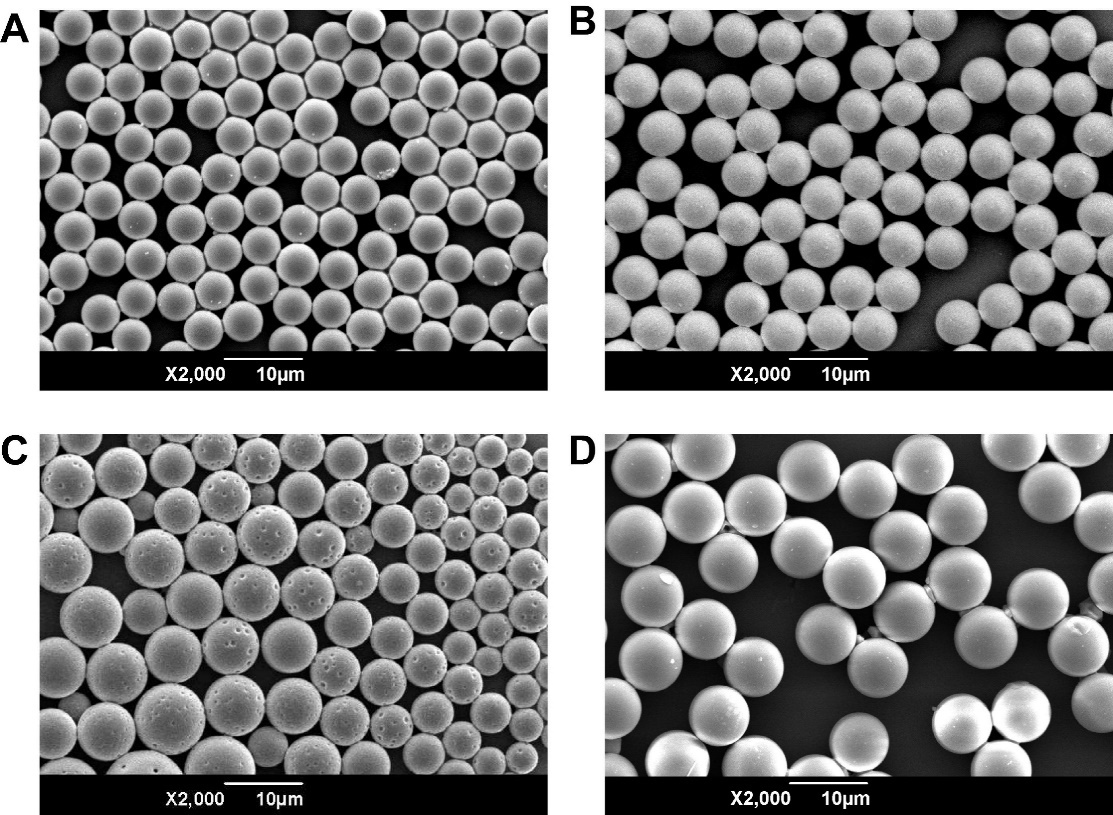 |
| --- |
| **Figure S1:** Scanning Electron Microscopy images of **(A)** PS, **(B)** PS-NH_2_, **(C)** PLGA, and **(D)** SiO_2_ microparticles. |

| 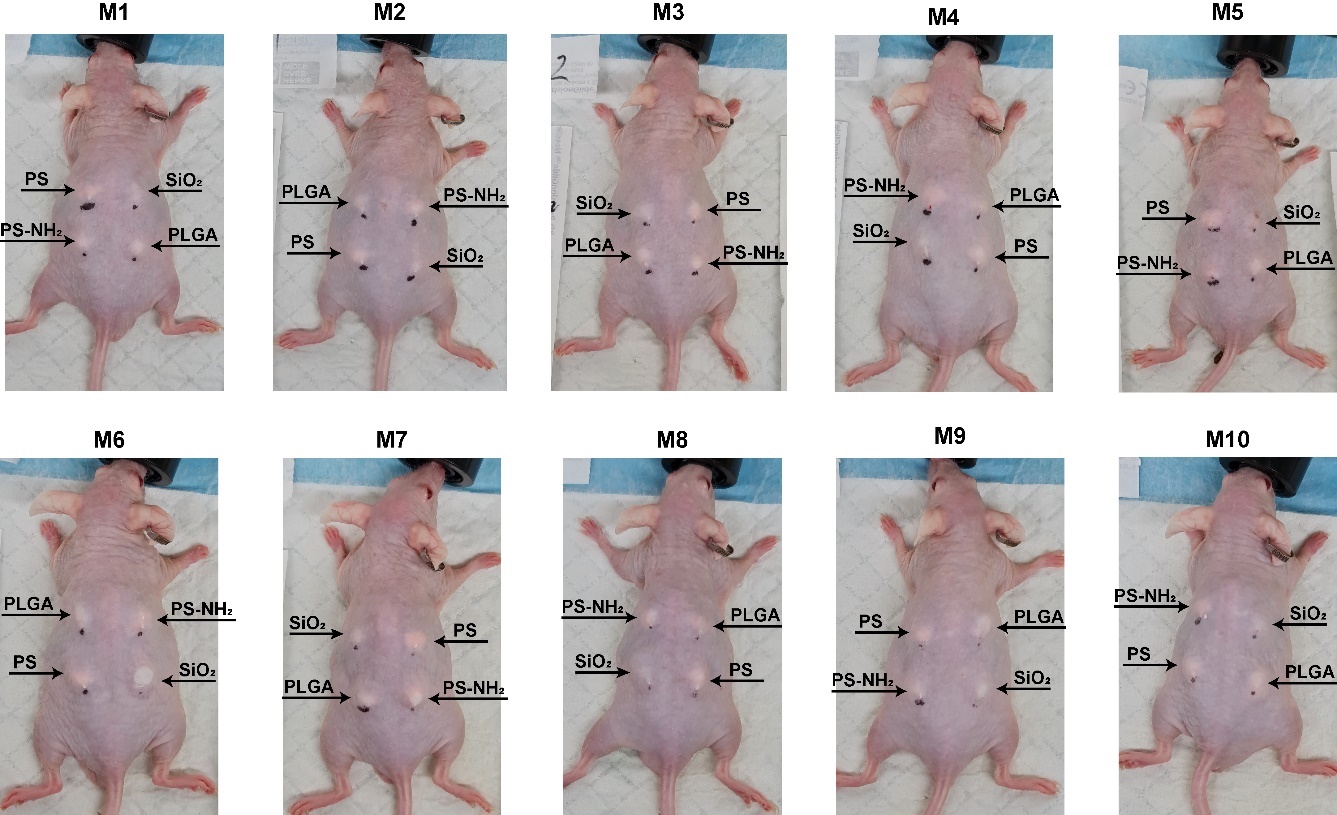 |
| --- |
| **Figure S2: Experimental design shown on representative photographs of SKH1-E mice indicating the spatial arrangement for 4 different microparticle formulations subcutaneously injected on the dorsal side of each mouse.** On each mouse, black arrows indicate 4 positions at which four different types of microparticles were injected. The injection positions of each microparticle formulation were alternated over all four positions possible on dorsal side of each mouse across N=10 biological repeats. All experiments followed this microparticle-injection map for their respective set of mice for each time point. Data from Figures 1 & 2 was obtained from 1 set of mice while Data from Figures 3 and 4 was obtained from another set of mice. |

| 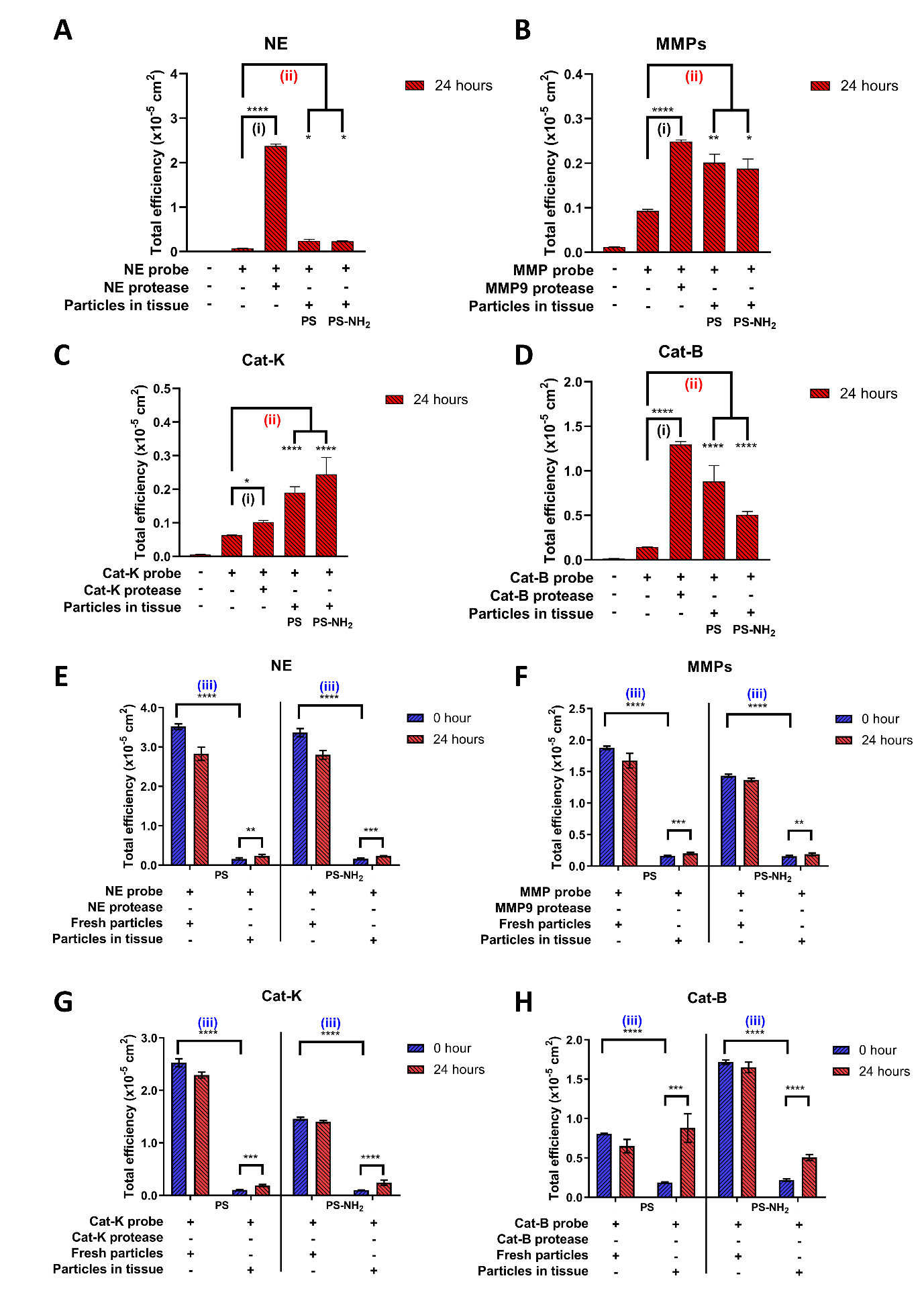 |
| --- |
| **Figure S3: Activation of imaging probes in the presence of their corresponding proteases or retrieved subcutaneous tissues containing subcutaneously injected microparticles.** Commercial imaging probes were activated by their corresponding proteases, **(A)** Neutrophil Elastase, **(B)** MMPs, (**C)** Cathepsin-K, and **(D)** Cathepsin-B, indicated by the increase in fluorescent signal 24 hours after imaging probes were mixed with their proteases (comparison **(i)** in panels **A, B, C** and **D)**. Similar increase in signals was observed when these imaging probes were incubated for 24 hours with retrieved microparticle-containing subcutaneous tissues, demonstrating the *ex vivo* activity of the proteases in the retrieved tissues (comparison **(ii)** in panels **A, B, C** and **D)**. When the imaging probes of **(E)** Neutrophil Elastase, **(F)** MMPs, (**G)** Cathepsin-K, and **(H)** Cathepsin-B were mixed with fresh PS or PS-NH_2_ particles suspended in PBS, signals artifact were detected immediately (0 hour) after their contact; but these signal artifacts were significantly lower after injected microparticles in retrieved subcutaneous tissues were exposed to the same probes (0 hour) (comparison **(iii)** in panels **E, F, G** and **H)**. Data presented in all subfigures of this Figure S3 were collected from the same experiment. Data represents mean ± SD of N=6 for all samples. P-values were determined by one-way ANOVA with Tukey’s multiple comparison test for multiple comparison or non-paired 2 tailed t-test for the same group between 0 hour and 24 hours. (*). (**), (***), (****) and ns denote P<0.05, P < 0.01, P < 0.001, P < 0.0001 and non-significant, respectively. |

| 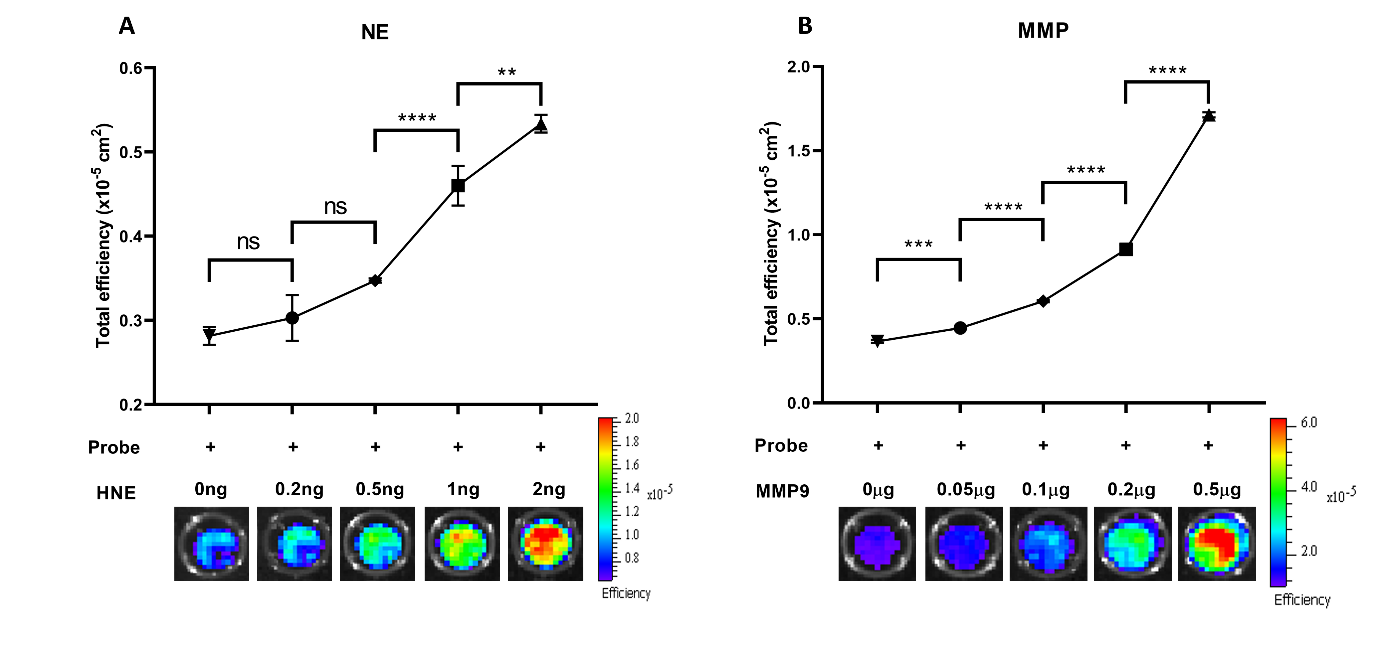 |
| --- |
| **Figure S4: *Quantitative correlation between fluorescent signal from activated probe and concentration of active protease*.** Fluorescent signals were detected in **(A)** NE probe and **(B)** MMP probe after they were incubated with different concentrations of active human recombinant NE or MMP9 for 24h. Data represents mean ± SD of n=3 for all samples. P-values were determined by one-way ANOVA with Tukey’s multiple comparison test. ns, (*), (**), (***) and (****) denote non significance, P < 0.05, P < 0.01, P < 0.001 and P < 0.0001, respectively. |

| 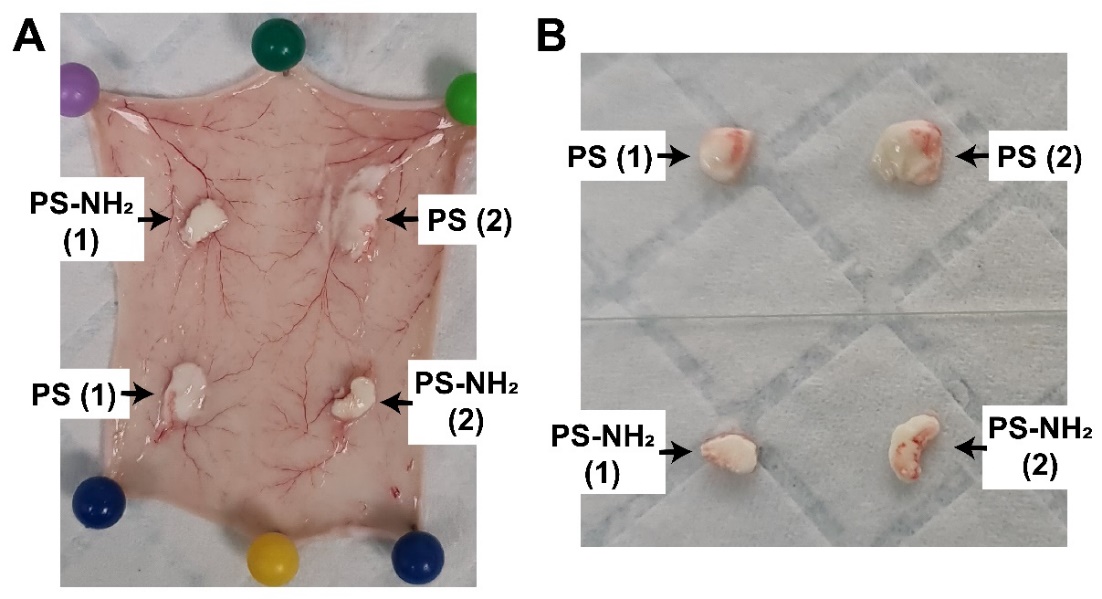 |
| --- |
| **Figure S5: Photographs of retrieved mouse dorsal skin containing subcutaneously injected microparticles on day 3 post-injection.** Representative images of **(A)** the dorsal skin containing subcutaneously injected microparticles and **(B)** subcutaneous tissues containing microparticles after retrieval. |

| 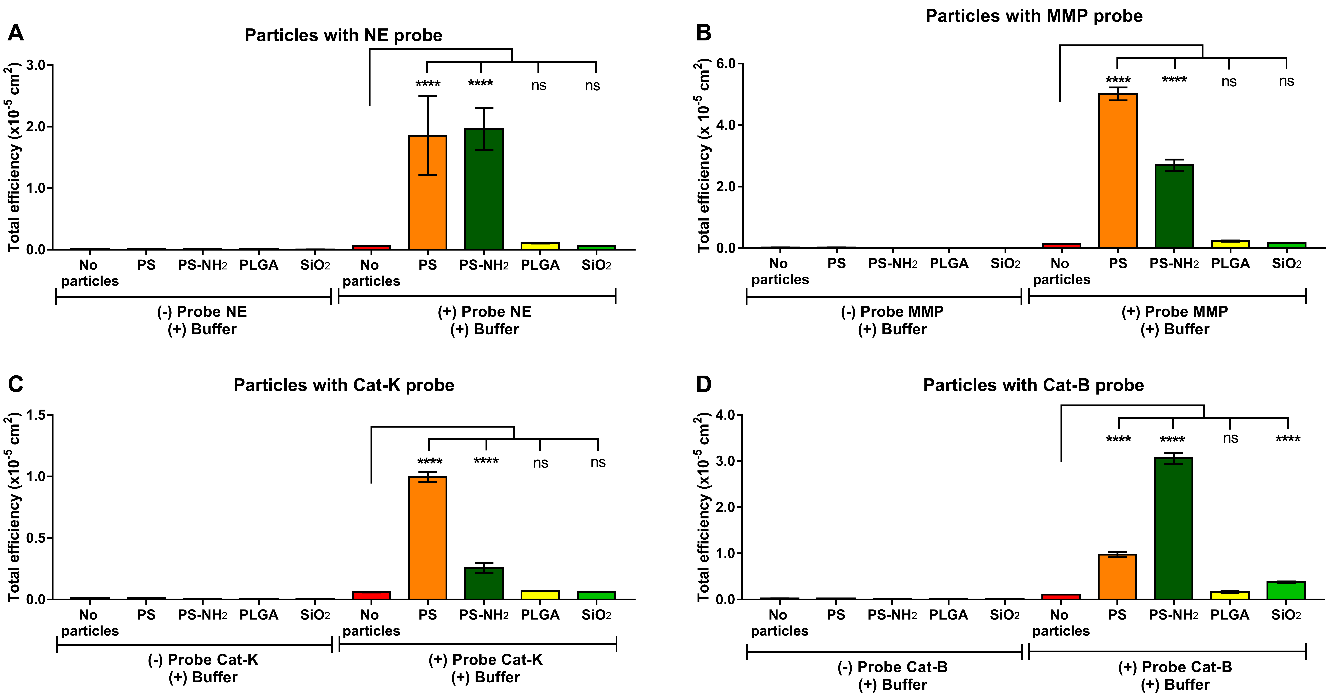 |
| --- |
| **Figure S6:** ***In vitro* interaction between imaging probes and microparticles.** Fluorescent signals were detected when PS or PS-NH_2_ microparticles were exposed to **(A)** NE probe, **(B)** MMP probe, **(C)** Cat-K probe, and **(D)** Cat-B probe without the presence of proteases. The interaction between either PS or PS-NH_2_ microparticles with imaging probes resulted in an artifact increase in fluorescent signal despite the absence of the proteases or retrieved tissue. Data represents mean ± SD of n=3 for all samples. P-values were determined by one-way ANOVA with Tukey’s multiple comparison test. (****) and “ns” denotes P < 0.0001 and non-significant, respectively. |

| 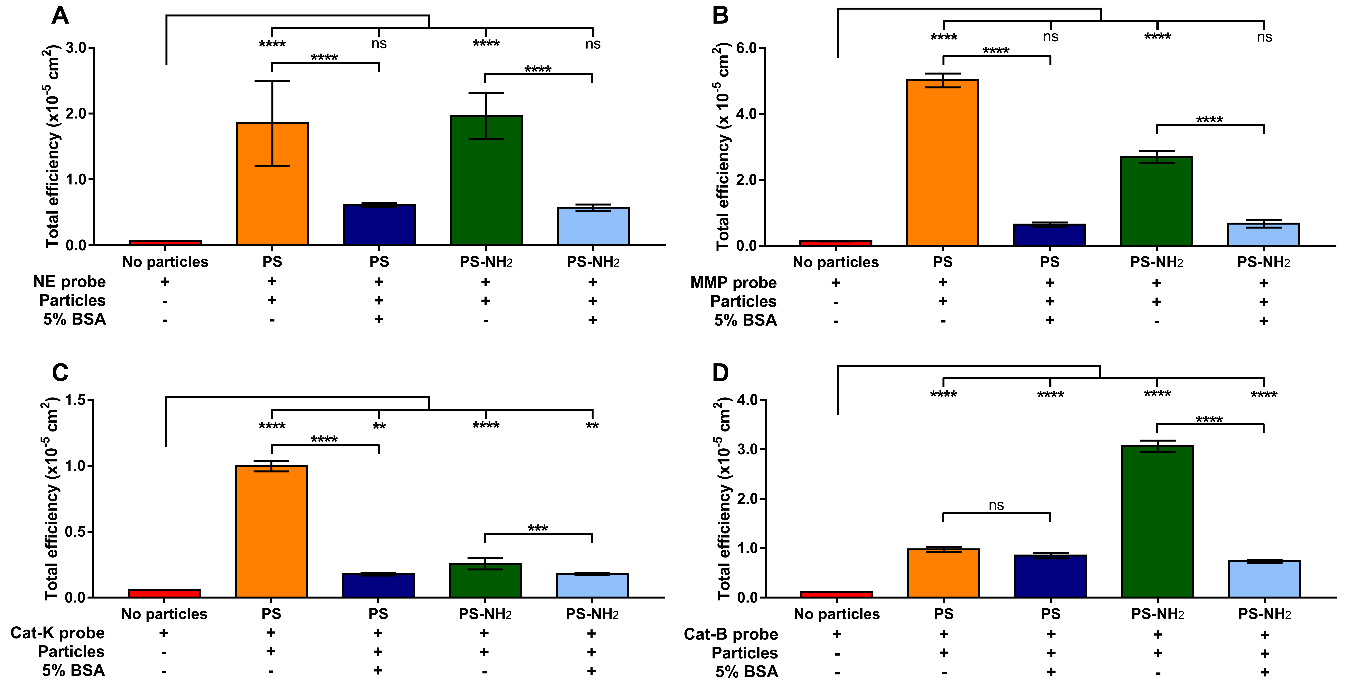 |
| --- |
| **Figure S7: BSA suppressed the fluorescent artifact from the interaction between particles with imaging probes.** Fluorescent signal artifacts from the interaction between PS or PS-NH_2_ microparticles with imaging probes for **(A)** NE, **(B)** MMP, **(C)** Cat-K, and **(D)** Cat-B were diminished in the presence of 5% BSA. The data represents mean ± SD of n=3 for all samples. P-values were determined by one-way ANOVA with Tukey’s multiple comparison test. (**), (***), (****) and ns denote P < 0.01, P < 0.001, P < 0.0001 and non-significant, respectively. Data of Figure S6 and this figure were collected from the same experiment. The data demonstrating fluorescent signal artifacts arising from interaction between PS or PS-NH_2_ microparticles with imaging probes in this figure were the same data presented in Figure S6. |

| 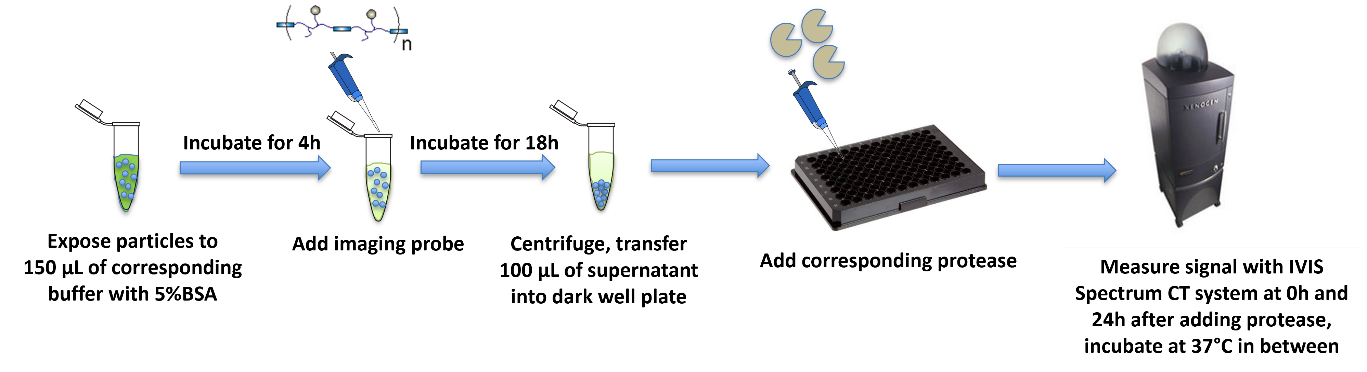 |
| --- |
| **Figure S8A. *In vitro* experimental procedure to quantitatively evaluate adsorption of each imaging probe by different materials.** Each microparticle formulation was first exposed to BSA for 4 hours to simulate *in vivo* protein adsorption*.* The imaging probe was then added into the particle suspension and incubated for 18 hours, which were similar to the time duration that the probe was *in vivo* prior to acquisition of fluorescent signal. After the microparticles (and in principle, probe hypothetically adsorbed on the particles, if any) were removed by centrifugation, the protease corresponding to the imaging probe was added to the supernatant. The fluorescent signal from the protease-containing supernatant was measured immediately and after 24h following protease addition, with this supernatant being incubated at 37°C in between these two timepoints. Hypothetically, if differential probe-material adsorption occurred due to the different material types, the removed microparticles with any adsorbed dye would result in differential concentrations of the probe remaining in the supernatant and subsequently, variation in the measured fluorescent signals from these supernatant samples after the corresponding protease was added. |

| 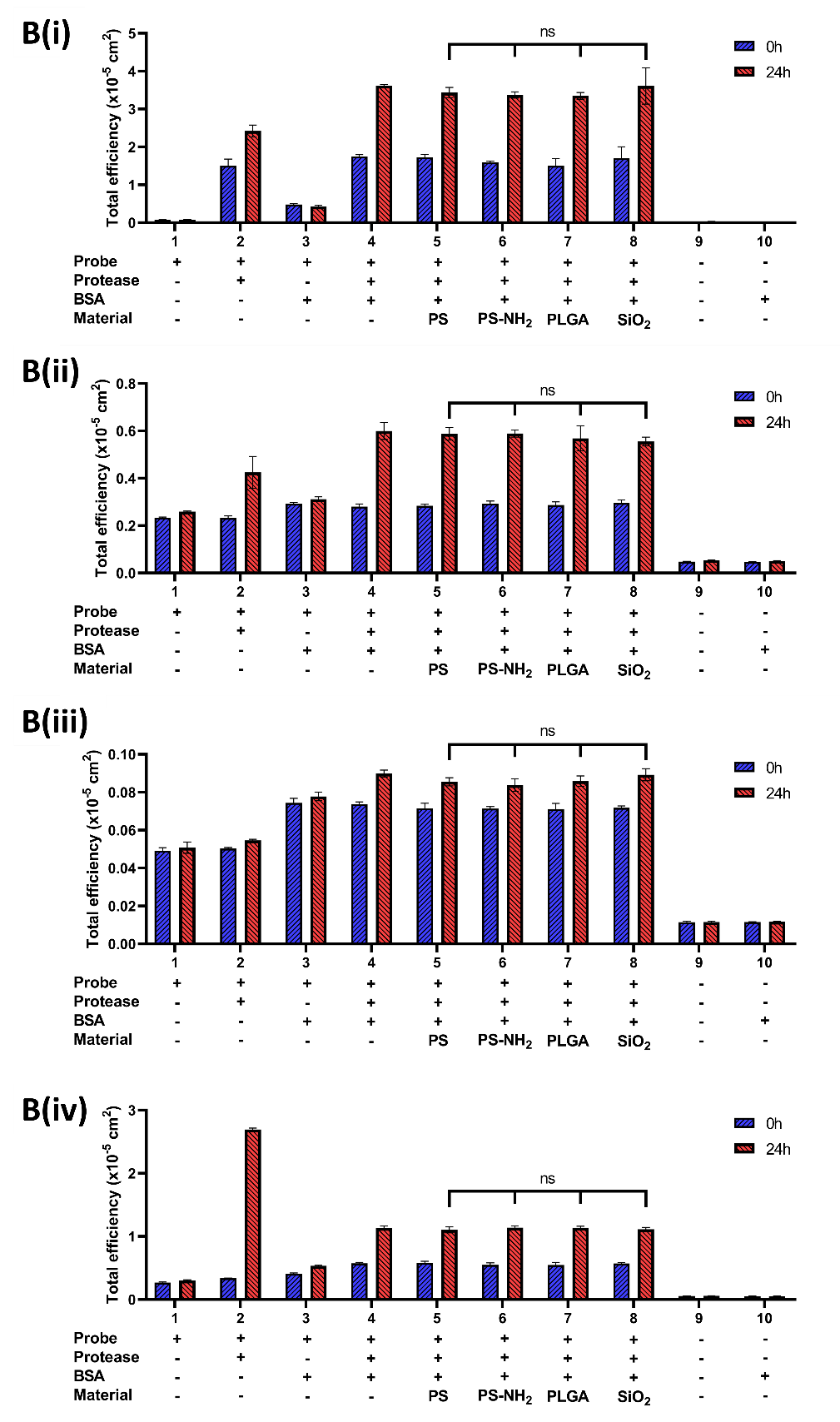 |
| --- |
| **Figure S8B: *In vitro* activation of imaging probe added to supernatant with prior exposure to BSA-coated microparticles.** Fluorescent signals were detected in (i) NE probe, (ii) MMP probe, (iii) Cat-K probe, and (iv) Cat-B probe after microparticles were removed and their corresponding proteases were introduced. There was no significant difference he fluorescent signals measured from the protease-containing supernatant with prior exposure to different materials 24h after the probes were activated and thus confirming that there was no differential adsorption of each imaging probe by the four microparticle formulations evaluated. Data represents mean ± SD of n=3 for all samples. P-values were determined by one-way ANOVA with Tukey’s multiple comparison test. “ns” denotes no significance. |

| 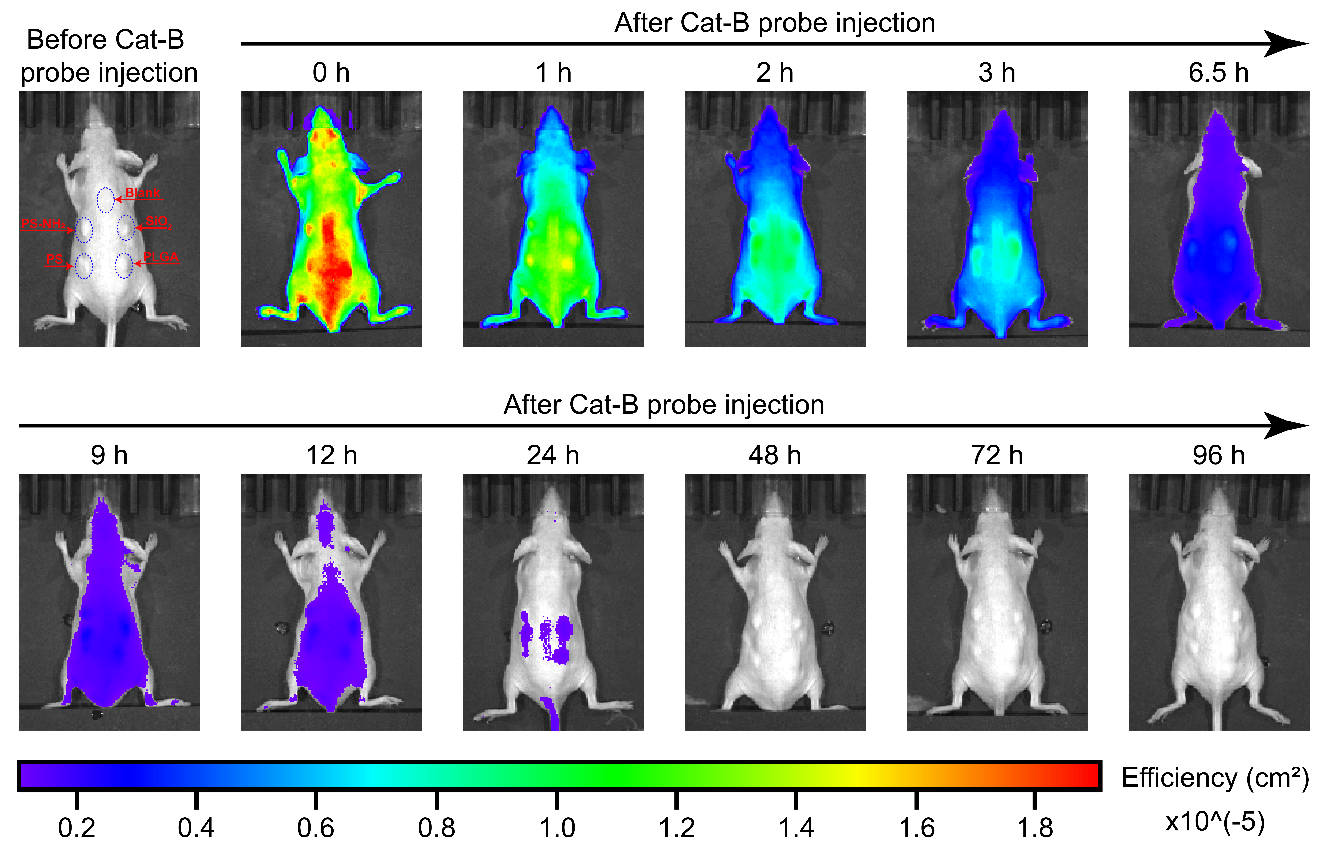 |
| --- |
| **Figure S9: Distribution and clearance kinetics of the Cat-B-activatable imaging probe in SKH1-E mice with microparticles subcutaneously injected on their dorsal region.** The signals faded rapidly in all locations on the animal body including the sites of microparticle injection as well as the blank spots that were not injected with microparticles. |

| 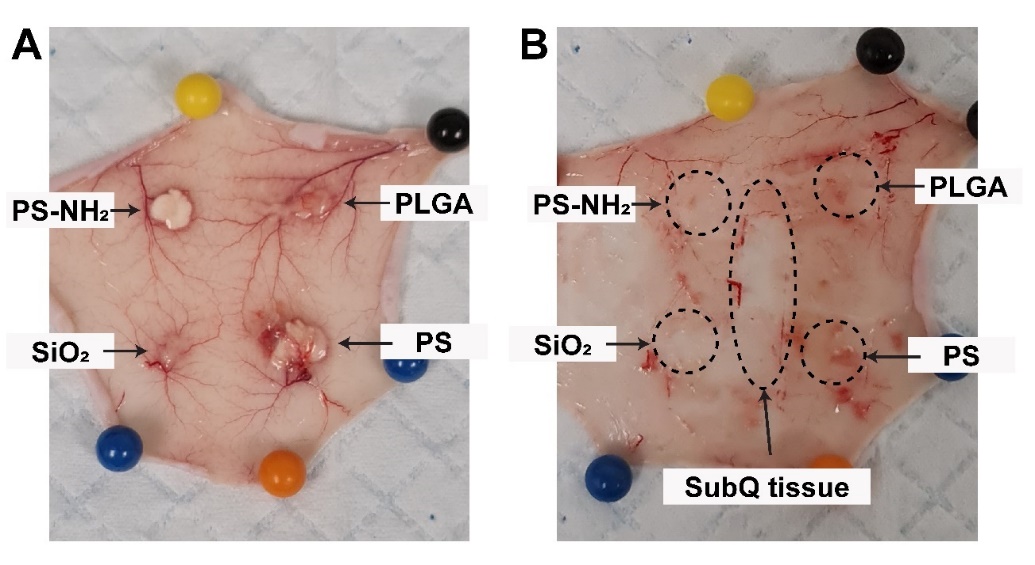 |
| --- |
| **Figure S10: Photographs of retrieved mouse dorsal skin containing subcutaneously injected microparticles on day 9 post-injection.** Representative images of the dorsal skin **(A)** before and **(B)** after retrieval of subcutaneous tissue layer without (SubQ tissue) or with injected microparticles (PS, PS-NH_2_, PLGA, and SiO_2_). |

| 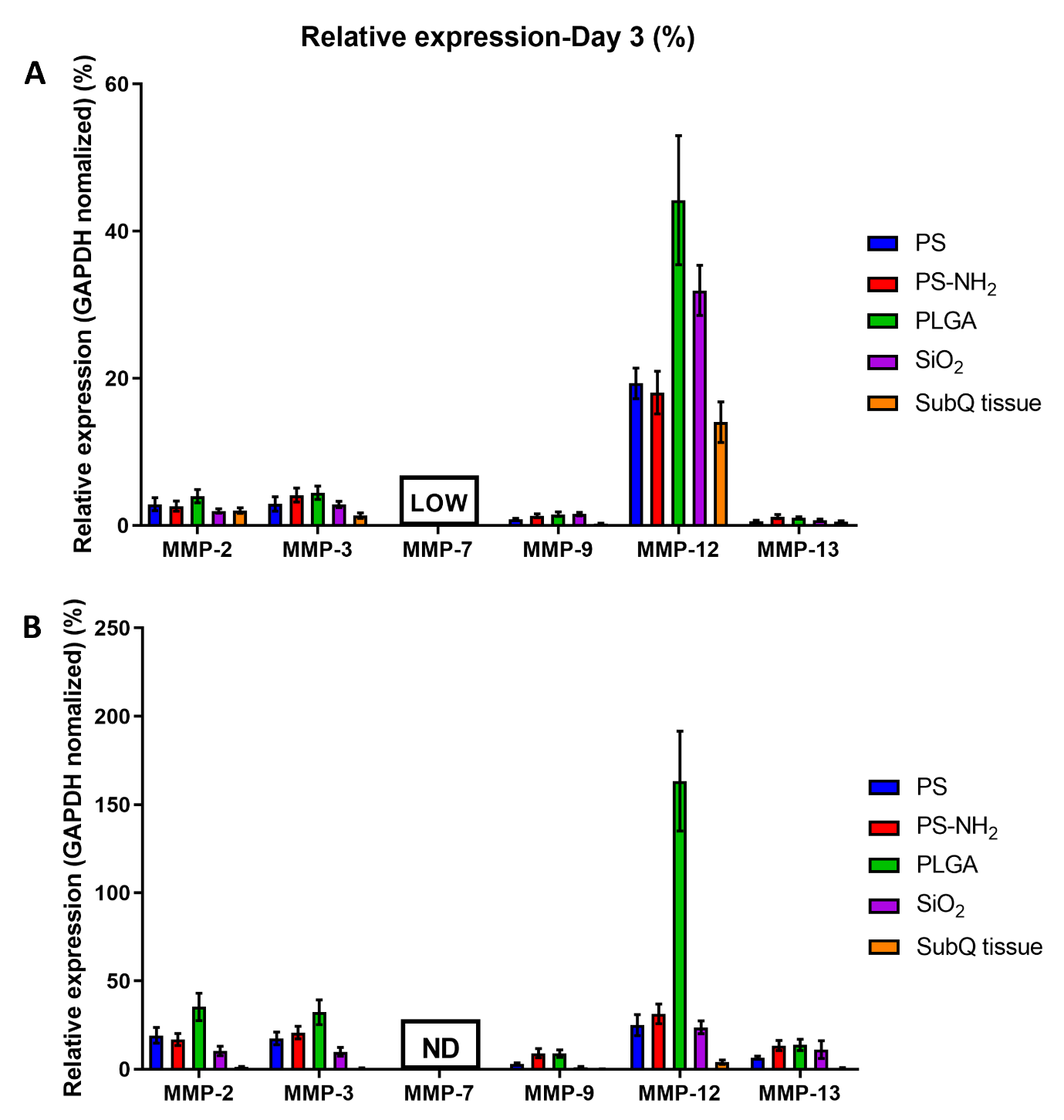 |
| --- |
| **Figure S11: mRNA expression of MMPs genes in retrieved subcutaneous tissues containing PS, PS-NH_2_, PLGA, and SiO_2_ microparticles (A) 3 days and (B) 9 days after their injection in SKH1-E mice.** Relative expression level for MMP-7 mRNA was lower than 0.01% for all materials at day 3 (Low) and it was not detectable at day 9 (ND) so it was excluded in subsequent analysis. Data represents mean ± SEM of N=10 for most samples, except for PS with N=9. |

| 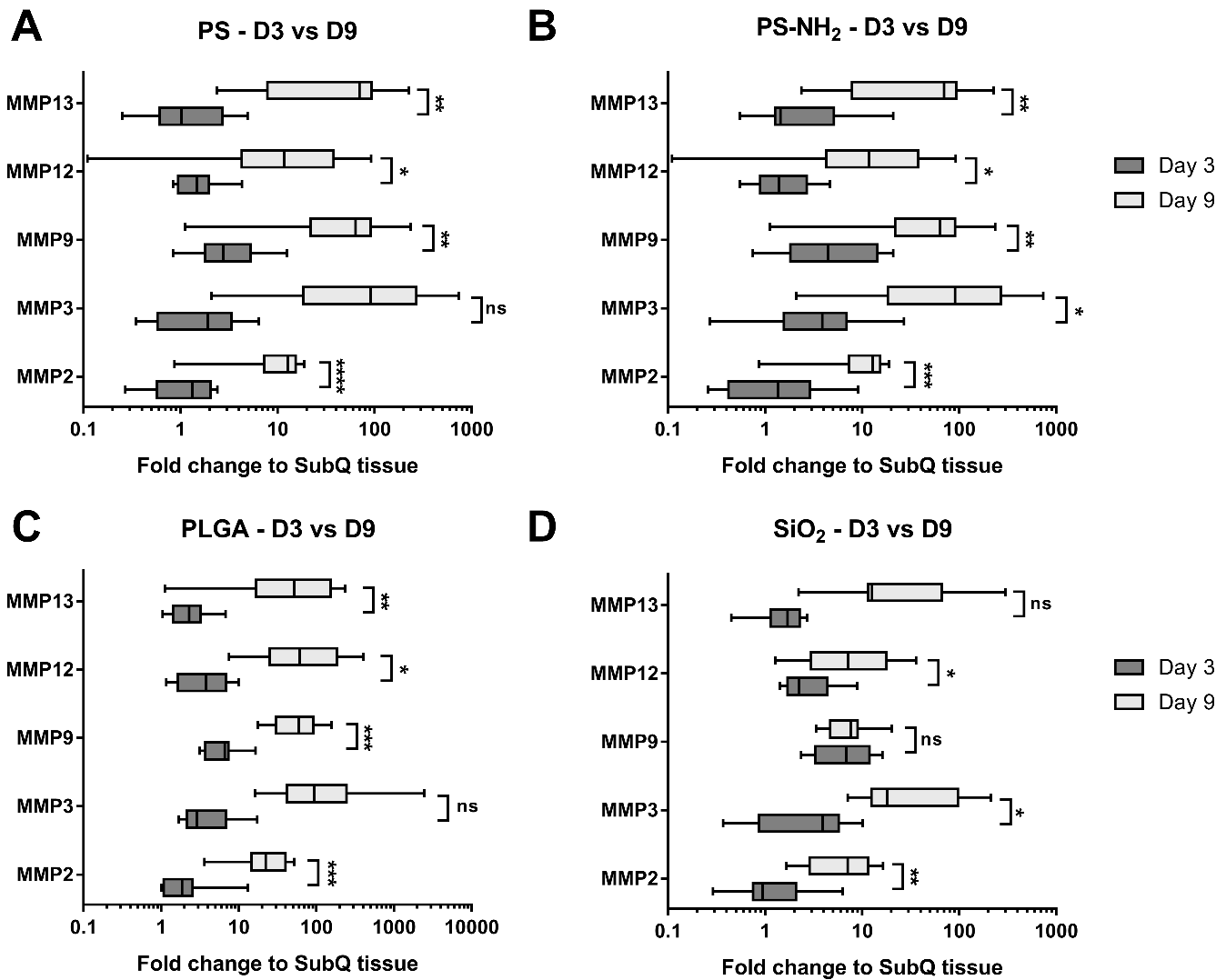 |
| --- |
| **Figure S12: mRNA expression of proteases of MMPs family in retrieved subcutaneous tissue containing injected microparticles compared to blank subcutaneous tissue**. **(A)** PS, **(B)** PS-NH_2_, **(C)** PLGA, and **(D)** SiO_2_ microparticles after injection 3 days and 9 days. mRNA expressions of most selected MMPs genes at the microparticle injection sites were increased on day 9 compared to those on day 3. Boxes represent medians ± IQR (interquartile range). Whiskers represent the minimum and maximum observations. P-values were determined by paired 2 tailed t-test. ns denotes non-significance. (*), (**), and (***) denote P < 0.05, P < 0.01, and P < 0.001, respectively. |

| 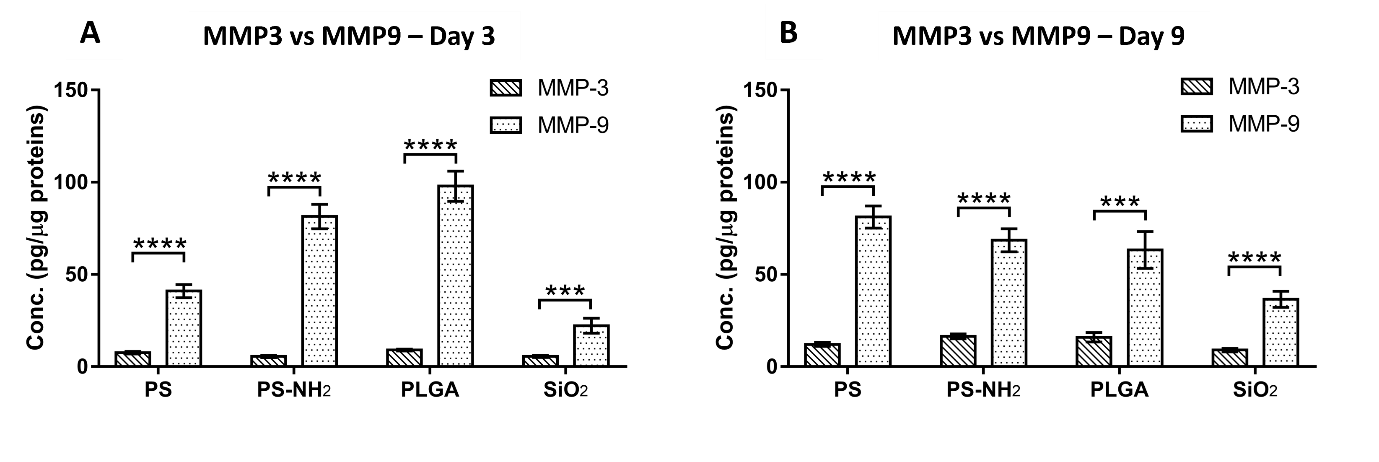 |
| --- |
| **Figure S13: Comparison of protein MMP-3 and MMP-9 expression on day 3 (A) and day 9 (B).** Total amount of MMP-3 protein was significantly lower than the total amount of MMP-9 protein in all material-containing tissues. Error bar represents mean ± SEM of n=10. P-values were determined by paired 2 tailed t-test. (*), (**), (***) and (****) denote P < 0.05, P < 0.01, P < 0.001 and P < 0.0001, respectively. |

| 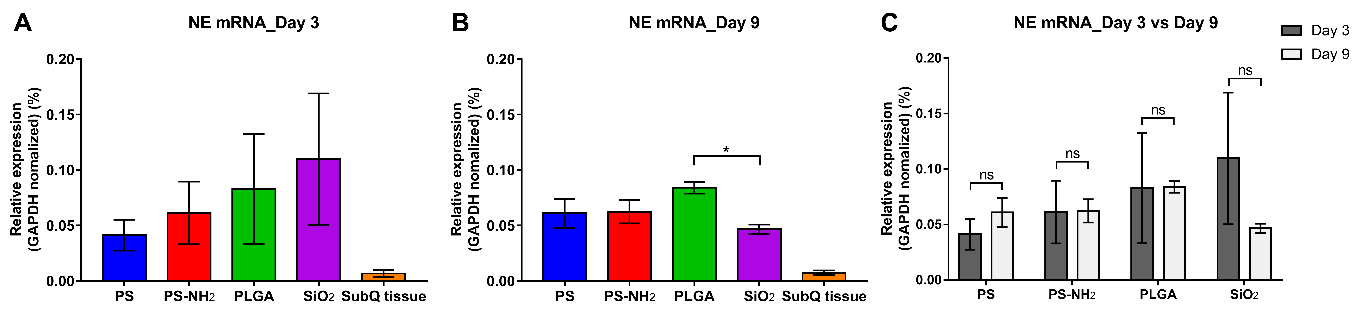 |
| --- |
| **Figure S14: mRNA expression of Neutrophil Elastase from retrieved subQ tissue containing injected materials on (A) Day 3, (B) Day 9, and (C) comparison of Day 3 vs. Day 9 (NE)**. All gene expression data were normalized against GAPDH. Data represents mean ± S.E.M of N=4, except N=3 for PS-Day 3. P-values of **(A)** and **(B)** were determined by one-way ANOVA with Tukey’s multiple comparison test. P-values of **(C)** were determined by non-paired 2 tailed t-test. (*), (**), and ns denote P < 0.05, P < 0.01, and non-significance, respectively. One data point from the retrieved tissue containing injected PS microparticles on day 3 was removed because the Grubb’s outlier test identified it as an outlier (p < 0.01). |

| 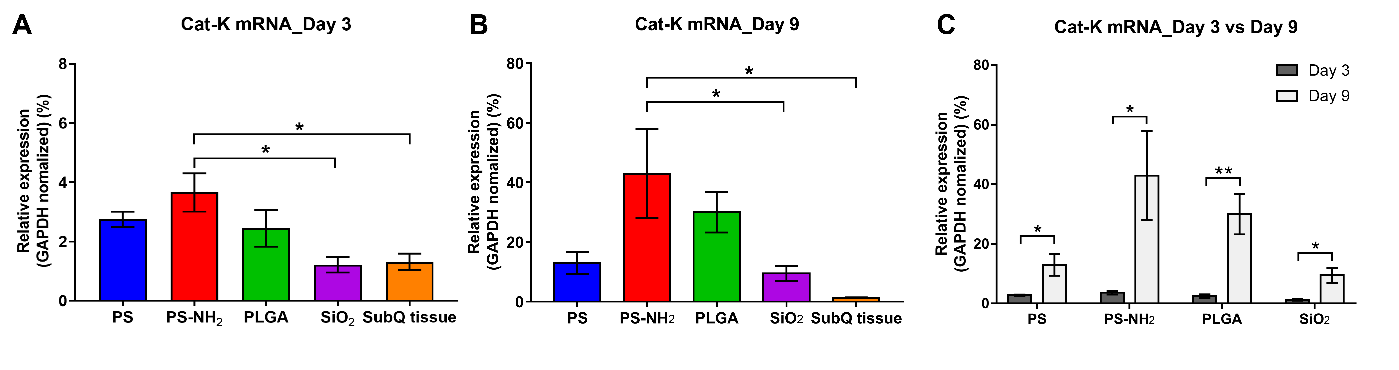 |
| --- |
| **Figure S15: mRNA expression of Cat-K from retrieved subQ tissue containing injected materials on (A) Day 3, (B) Day 9, and (C) comparison of Day 3 vs. Day 9.** All gene expression data were normalized against GAPDH. Data represents mean ± S.E.M of N=4. P-values of **(A)** and **(B)** were determined by one-way ANOVA with Tukey’s multiple comparison test. P-values of **(C)** were determined by non-paired 2 tailed t-test. (*), (**), and ns denote P < 0.05, P < 0.01, and non-significance, respectively. |

| **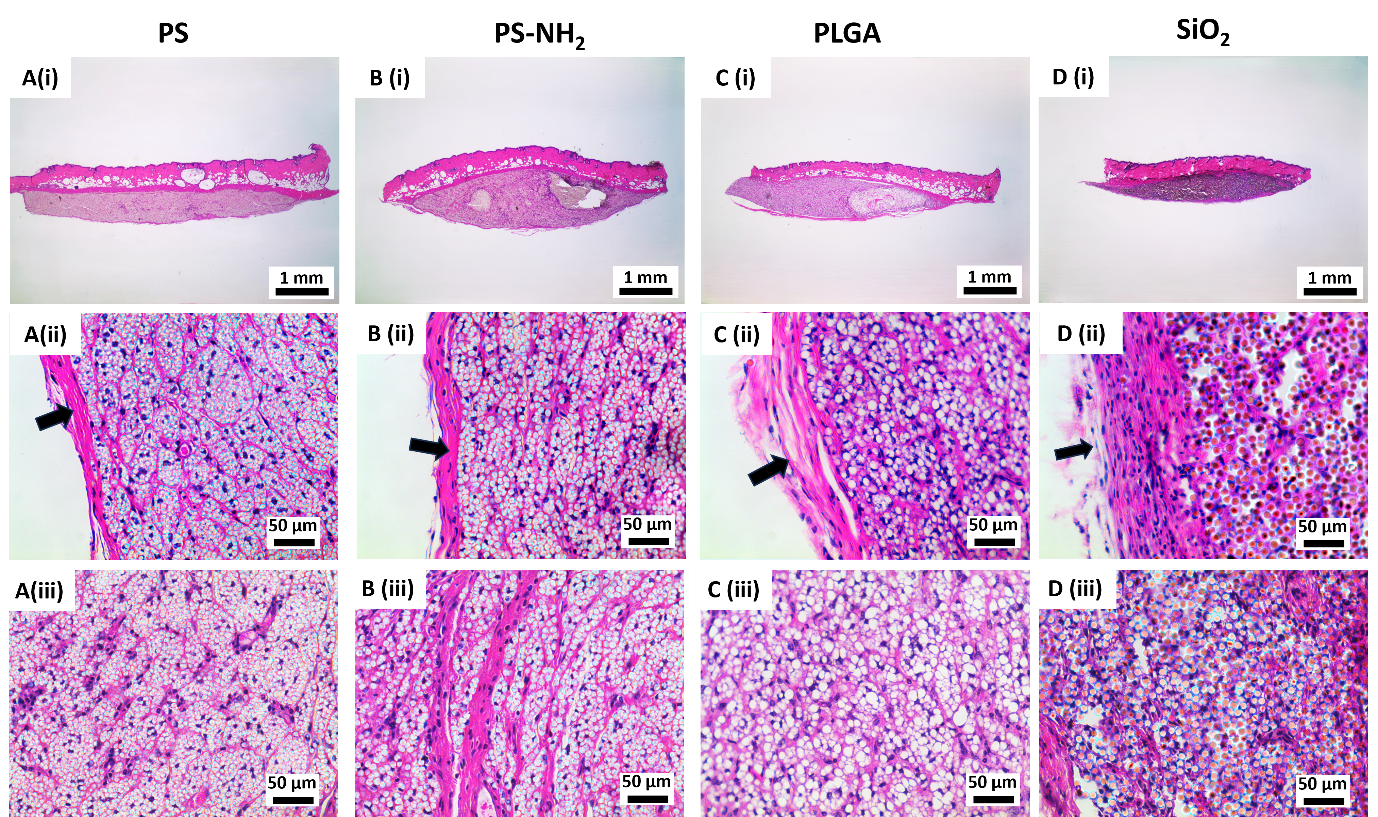** |
| --- |
|  |
| **Figure S16: Hematoxylin and Eosin histological sections of excised dermal and subQ tissues containing subcutaneously injected microparticles (5mg/injection) retrieved from SKH1-E mice on day 28 after microparticle injection.** (i) Photos of tissue sections containing **(A)** PS, **(B)** PS-NH2, **(C)** PLGA, or **(D)** SiO2 microparticles at 2X magnification. (ii) Photos of tissue sections containing **(A)** PS, **(B)** PS-NH2, **(C)** PLGA, or **(D)** SiO2 microparticles at 10X magnification. Black arrows indicate the multiple fibrotic cellular layers at the outer edge of the aggregated particle mass. (iii) Photos of tissue sections containing **(A)** PS, **(B)** PS-NH2, **(C)** PLGA, or **(D)** SiO2 microparticles located at the interior region of the aggregated particle mass. Scale bars indicate 1mm in **A**(i)-**D**(i) and indicate 50 µm in **A**(ii)-**D**(ii) and **A**(iii)-**D**(iii). |

| **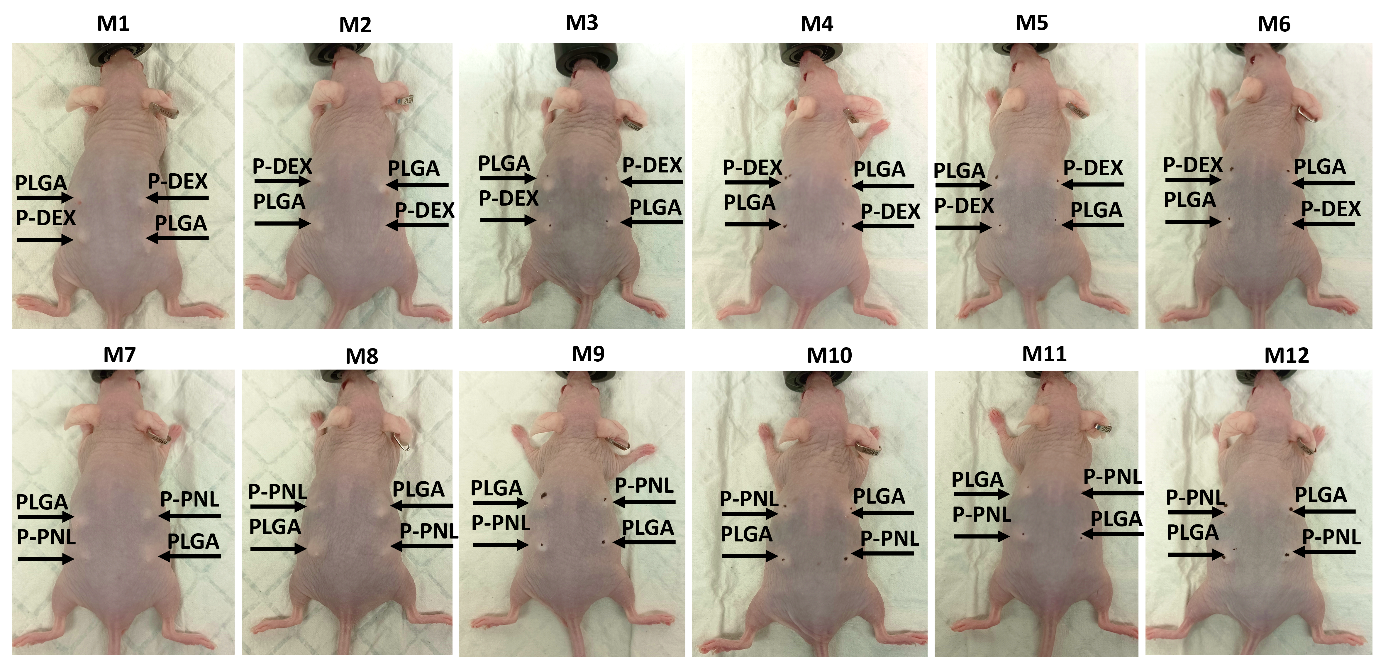** |
| --- |
| **Figure S17: Experimental design shown on representative photographs of SKH1-E mice indicating the spatial arrangement for compound-loaded PLGA formulations subcutaneously injected on the dorsal side of each mouse.** On each mouse, black arrows indicate 4 positions at which two formulations (PLGA and either P-DEX or P-PNL) were injected. The injection positions of each microparticle formulation were alternated over all four positions possible on dorsal side of each mouse across N=12 biological repeats. |

| 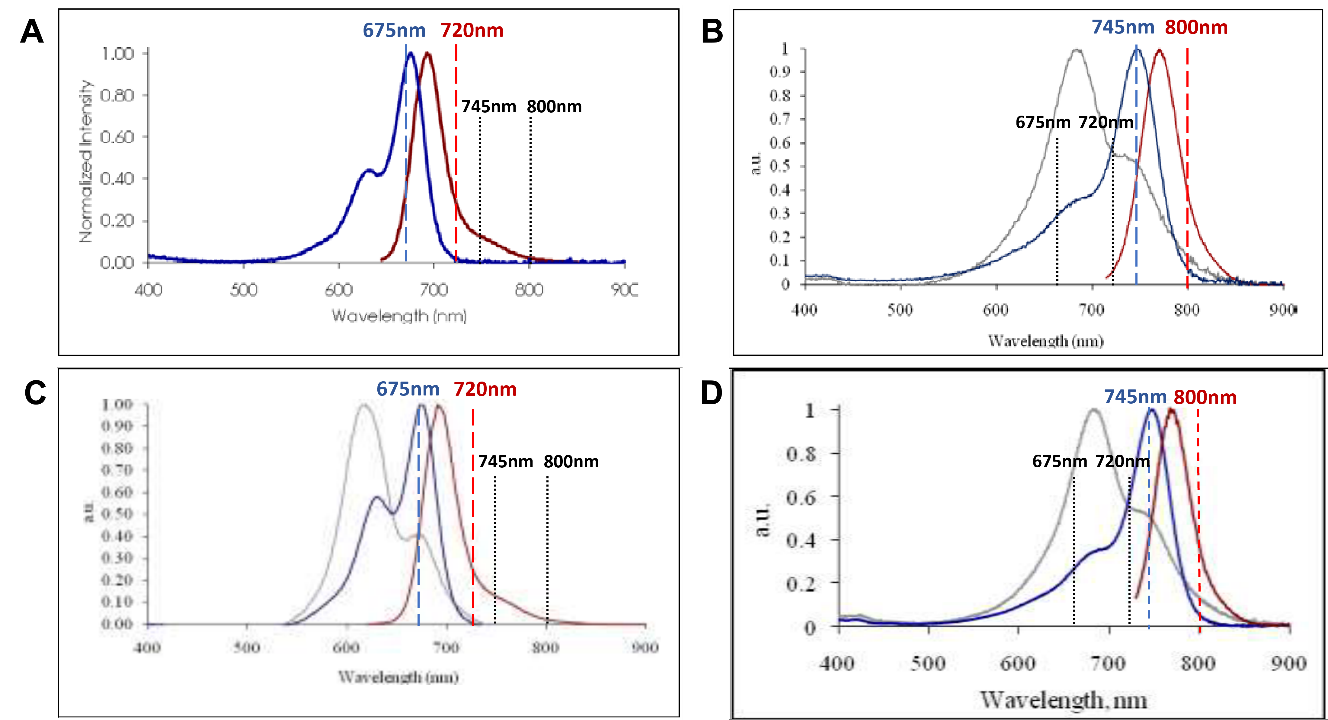 |
| --- |
| **Figure S18: Fluorescence spectra of protease-activatable probes.** Excitation (blue), emission (red), and normalized absorbance (grey) of **(A)** Neutrophil Elastase 680 FAST, **(B)** MMPSense 750 FAST, **(C)** Cat K 680 FAST, and **(D)** Cat B 750 FAST in 1x PBS. For each probe, the excitation and emission wavelengths were indicated by the long vertical blue and red dashed lines, respectively. The short dotted black lines indicated the excitation and emission wavelength of its co-injected pairing probe. Emissions of Neutrophil Elastase FAST (A) and CatK (C) FAST are negligible at 800nm; MMPsense (B) and CatB FAST (D) have minimal emission at 720nm. Since Neutrophil Elastase probe was paired with MMPSense, while CatK FAST and CatB FAST were paired, there is negligible signal interference between the probes used in pair. Subfigures A, B, C, and D were reproduced in full from references [2], [3], [4], and [5], respectively with permission. |

**References for SI**

1. Truong THA, Mothe SR, Min JL, Tan HM, Jackson AW, Nguyen DT, et al. Immuno‐modulatory effects of microparticles formulated from degradable polystyrene analogue. Macromolecular Bioscience. 2022;22(7):2100472.

2. PerkinElmer I. TECHNICAL DATA SHEET - Neutrophil Elastase 680 FAST™.

3. PerkinElmer I. TECHNICAL DATA SHEET - MMPSense™ 750 FAST.

4. PerkinElmer I. TECHNICAL DATA SHEET - Cat K™ 680 FAST.

5. PerkinElmer I. TECHNICAL DATA SHEET - Cat B 750 FAST™.
